# Fluted-point technology in Neolithic Arabia: An independent invention far from the Americas

**DOI:** 10.1371/journal.pone.0236314

**Published:** 2020-08-05

**Authors:** Rémy Crassard, Vincent Charpentier, Joy McCorriston, Jérémie Vosges, Sofiane Bouzid, Michael D. Petraglia

**Affiliations:** 1 CNRS, USR 3141 CEFAS, Centre Français d’Archéologie et de Sciences Sociales, Kuwait City, Kuwait; 2 CNRS, UMR 5133 Archéorient, Maison de l’Orient et de la Méditerranée, Lyon, France; 3 Inrap & CNRS, UMR 7041 ArScAn Archéologie et Sciences de l’Antiquité, MSH Mondes, Nanterre, France; 4 Department of Anthropology, The Ohio State University, Columbus, OH, United States of America; 5 Department of Archaeology, Max Planck Institute for the Science of Human History, Jena, Germany; 6 Human Origins Program, National Museum of Natural History, Smithsonian Institution, Washington, DC, United States of America; 7 School of Social Science, University of Queensland, Brisbane, Australia; University at Buffalo - The State University of New York, UNITED STATES

## Abstract

New World archaeologists have amply demonstrated that fluted point technology is specific to Terminal Pleistocene American cultures. Base-fluted, and rarer tip-fluted, projectile points from the Americas have been well-documented by archaeologists for nearly a century. Fluting is an iconic stone tool manufacturing method and a specific action that involves the extraction of a channel flake along the longitudinal axis of a bifacial piece. Here we report and synthesize information from Neolithic sites in southern Arabia, demonstrating the presence of fluting on a variety of stone tool types including projectile points. Fluted projectile points are known from both surface sites and stratified contexts in southern Arabia. Fluting technology has been clearly identified at the Manayzah site (Yemen) dating to 8000–7700 cal. BP. Examination of fluted points and channel flakes from southern Arabia enable a reconstruction of stone tool manufacturing techniques and reduction sequences (*chaines opératoires*). To illustrate the technological similarities and contrasts of fluting methods in Arabia and the Americas, comparative studies and experiments were conducted. Similarities in manufacturing approaches were observed on the fluting scars of bifacial pieces, whereas technological differences are apparent in the nature and localization of the flute and, most probably, the functional objective of fluting in economic, social and cultural contexts. Arabian and American fluted point technologies provide an excellent example of convergence of highly specialized stone tool production methods. Our description of Arabian and American fluting technology demonstrates that similar innovations and inventions were developed under different circumstances, and that highly-skilled and convergent production methods can have different anthropological implications.

## Introduction: The fluting method and its aims

### Fluting as a flintknapping method

Fluting is a flintknapping process or a “method” (*sensu* [1]:30) consisting of the removal of one or more long and flat flakes along the central axis of a generally thin, leaf-shaped bifacial blank. Fluted items are made from knappable, usually cryptocrystalline, rocks including flint, chert, quartzite, obsidian, chalcedony and crystal quartz. Fluting is applied from one of the two extremities of a bifacial piece, i.e., either executed at the base or from the tip. Rarer examples show bi-fluting from both the base and tip. In North America, the aim of fluting is to thin the bifacial piece, and more specifically, to produce a hafting zone on both faces of the piece, the obtained product of which is often used as a projectile point, such as an arrowhead, dart or spear tip [2].

**Fig 1:**
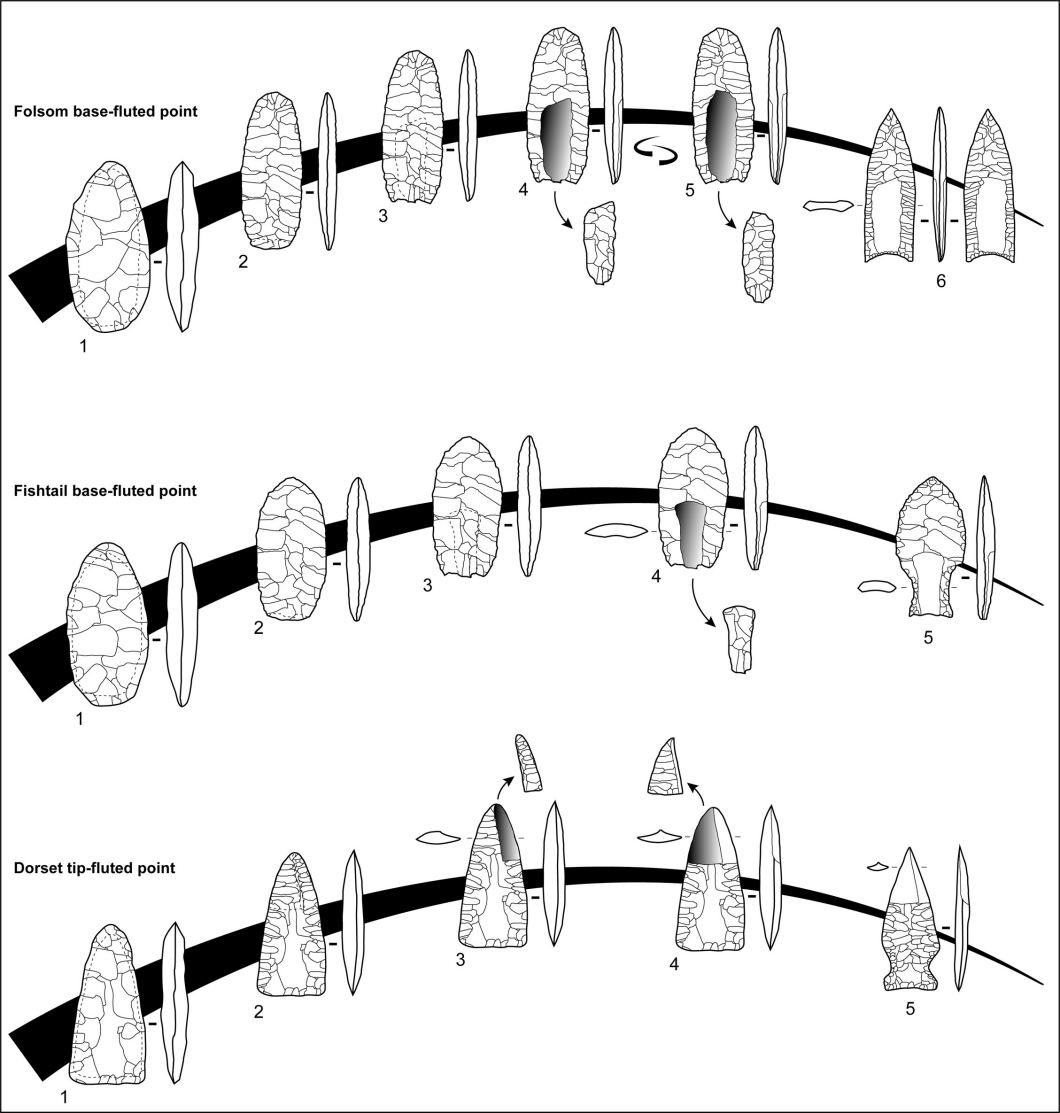
Theoretical *chaines opératoires* of fluted projectile points from the Americas. Folsom base-fluted point, 1 and 2: bifacial preform reduction, 3: preparation of fluting platform, 4 and 5: fluting along both faces, 6: final fluted point. Fishtail base-fluted point, 1 and 2: bifacial preform reduction, 3: preparation of fluting platform, 4: fluting along one face, 5: final fluted point. Dorset tip-fluted point, 1 and 2: bifacial preform reduction, 3 and 4: fluting of two overflowing flakes from the tip, 5: final fluted point.

The flake obtained by the fluting removal is variably called a “channel flake” [3], a “fluting flake” [2] or a “fluting spall” [4]. It is recognizable as part of the fluting method, as it shows the scars of the previous bilateral removals of the bifacial piece. These scars are thus “cut” by the fluting flake, leaving only the negatives of the medial and/or distal parts of the previous bifacial removals (Fig 1). These channel flakes are waste products and not formal tools. The fluting scar can extend from a few millimeters in the case of a hinged fluting flake, to several centimeters if the flute reaches the terminal end of the bifacial piece.

**Fig 2:**
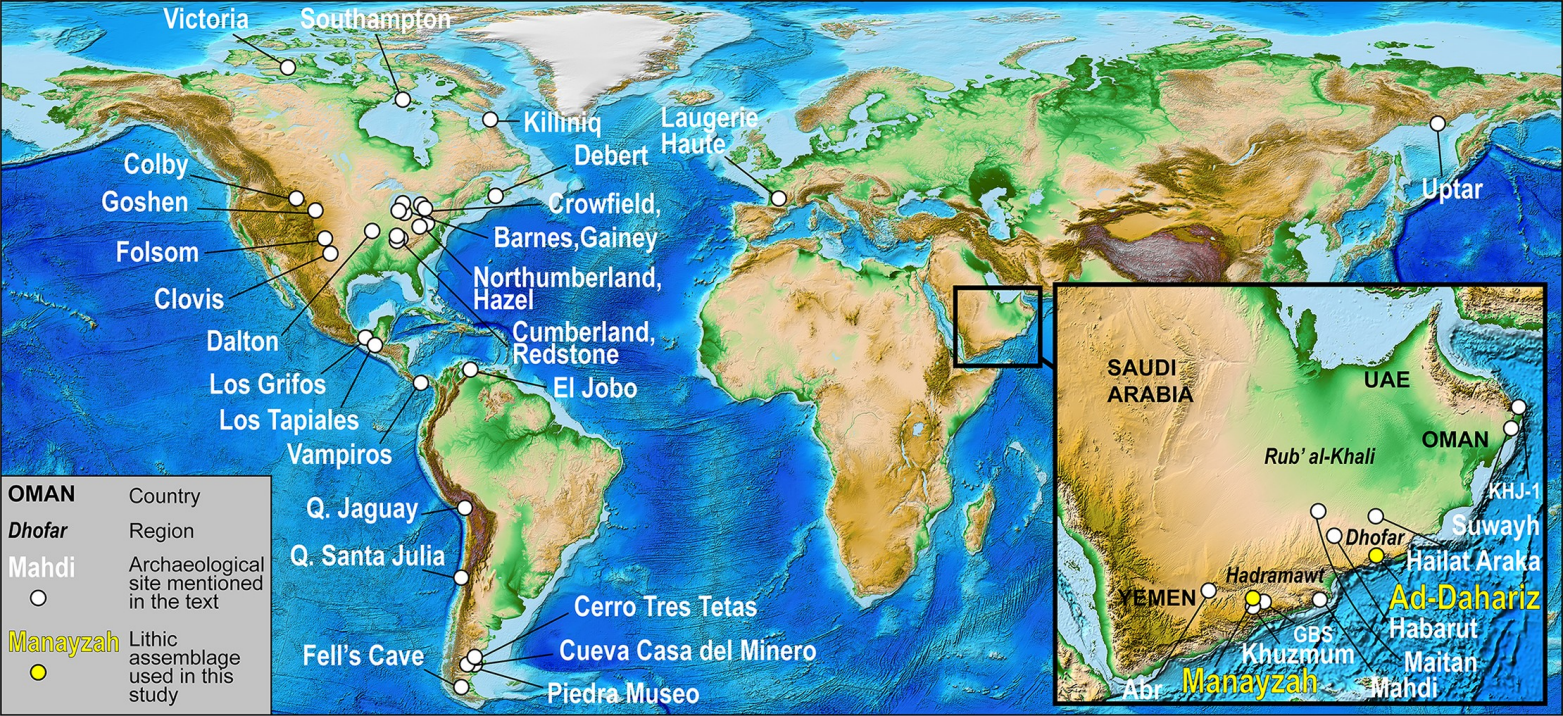
Map displaying key archaeological sites, including in southern Arabia (enlarged zone), North, Central and South America, France and Siberia. Modified from a NCEI world digital model, doi:10.7289/V5C8276M (source: https://www.ngdc.noaa.gov/mgg/global/global.html).

Known for nearly a century now, the fluting method is well-identified throughout the Americas, extending from the Arctic to Patagonia, and typically occurring in Terminal Pleistocene contexts. The relatively recent discovery of fluted points in the southern part of the Arabian Peninsula (Yemen, Oman and the United Arab Emirates; Fig 2) [5] indicates that Early Holocene populations of southern Arabia manufactured points employing the fluting method. Across the well-studied lithic industries of the prehistoric Near East and northern Africa, no other examples of fluted points are known. Indeed, fluting does not appear in other large areas of the Arabian Peninsula, such as in the Rub’ al-Khali Desert (Empty Quarter) and in the Nefud Desert of central and northern Saudi Arabia. The Arabian fluting method therefore appears to be a local innovation of southern Arabian groups, most probably originating in the central region of the southern extremity of the peninsula, where an arid landscape of highly incised limestone plateaus characterizes the provinces of Hadramawt and Mahra in eastern Yemen and Dhofar in western Oman (e.g. [6,7]). The Arabian and American examples may be readily differentiated based on the fact that the Arabian points have distinctive shapes and fluting does not systematically occur at the base of projectile points. Therefore, the aim of fluting in Arabia appears to be for a different purpose in comparison to the American examples, which primarily involves mounting a projectile point to a haft for functional reasons.

The aim in this article is to examine the alternate aims of fluting in the Americas and in Arabia. Here we describe the Arabian fluting method and fluting detachment techniques and their techno-typology. To better understand the nature of the fluting method in Arabia, we first briefly review fluting technology in the Americas and some rarer examples described from the Old Word. The evidence for fluting technology in Arabia will then be presented. The similarities and differences between American and Arabian fluting methods will then be highlighted. We aim to demonstrate that, in contrast to the situation in the Americas, the Arabian examples are likely made for sociocultural reasons rather than for functional uses.

### Why flute?

The question as to why prehistoric knappers chose to flute has been raised by scholars for decades without leading to a definitive answer [3,8–22]. Researchers have usually concluded that the aim of fluting was to thin points, allowing for ease in hafting [23–26]. Such a design indicates that the point was fit to a shaft with care and precision [3].

Though fluted pieces may have aided hafting, it has been noted that unfluted bifacial pieces were often thin enough for hafting, without requiring the risk to break or damage a complex piece on which the knapper worked skillfully. With this potential danger in mind, the rationale for fluting has remained somewhat unclear in terms of specific function. Extracting one or two elongated flakes along the axis of a point risked breaking the piece, as has been amply demonstrated in the archaeological record [12]. If the point survived the final knapping stage of fluting, a fluted point is sometimes more fragile and less useful; however, one study demonstrated that the fluted point base may have acted as a potential “shock absorber”, showing that these points could withstand physical stress compared against unfluted points [27]. Certain researchers have suggested that fluting lessened the weight of projectile points thereby aiding flight, while others have argued that the flute facilitated the ease of extraction of the spear point from a wound ([3]:7,[24]).

Comparisons with modern weaponry have led some to interpret the flute scar as a blood groove, as seen on modern metal hunting or fighting knives. Thus, the flute would have stimulated blood loss in the animal attacked, or would allow wounded prey to bleed more freely. This makes little sense, however, as the flute would be covered by the haft [2]. The use of poison has also been suggested, as the groove could have received such a substance, aiding in the hunting of megafauna, such as mammoths and mastodons (*Mammuthus primigenius*, *Mammuthus columbi*, *Mammut americanum*) [28,29]. Hemoglobin crystallization and red blood cell size analyses from residues on Beringian fluted projectile points [30] have demonstrated that a variety of mammals were hunted, including bison (*Bison bison*), sheep (*Ovis dalli*), bear (*Ursus arctos*), caribou (*Rangifer tarandus*), and musk ox (*Ovibos moschatus*). The use of poison is nevertheless not mandatory for hunting this range of species.

Experimental approaches reveal another potential non-utilitarian explanation. Fluting a point may be considered the pinnacle achievement for a craftsman. Today, flintknappers often consider that this operation is a significant achievement and a kind of “bravado,” or a successful demonstration of a high-risk undertaking ([2]:235). Fluting could therefore have involved the outward expression of a skill display beyond the application of merely functional or pragmatic roles [31], thereby serving “as much as a socio-cultural role as a techno-functional one” ([21]:39).

The wide range of interpretations summarized here show some level of uncertainty as to answering the question as to ‘why flute’. Though utilitarian and non-utilitarian interpretations have been put forward, it may also be the case that these explanations are not necessarily mutually exclusive but complementary. For American examples, it appears that most researchers center on the argument that the fluting method is used for facilitating hafting on projectile points, thereby emphasizing a functional role in hunting.

### Geographic distribution of the fluting method across the world

#### Fluting in the New World

The fluting method has been widely studied by American archaeologists (e.g. [32,33]; see the recent volume: [34]; see also [35,36] and the online Paleoindian Database of the Americas: http://pidba.utk.edu/). The first well-known discovery of fluted points was in 1926 near the town of Folsom, New Mexico. Found in association with the remains of extinct animals, the chipped stone projectile points included an example embedded in the ribs of an extinct species of bison [37,38]. Because other styles of ‘Folsom points’ were found in many parts of the New World, the term ‘fluted point’ was developed by Shetrone [39], who described bifacial points that had a longitudinal flute or groove extending from the base to a varying distance toward the tip on each face. Identified in 1935 at its eponymous site, ‘Clovis points’ were considered typologically distinct from Folsom points [40]. Clovis points were often longer, but with a shorter flute compared to Folsom points, where the flute scar is highly invasive on each face of the bifacial piece. Clovis points were therefore interpreted to be part of a distinct and early techno-complex in North America [33,41,42] and later found to date to a particular period of time, between ca. 13,400–12,900 years ago [43]. Coinciding with the Younger Dryas cooling event, a various range of fluted point types are now known to have developed as hunter-gatherer groups adapted to progressively more varied environments [22,44,45]. Post-Clovis fluted points were used by different populations up until ca. 10,000 years ago [46,47], or even later [48].

Folsom and Clovis lie at the heart of the identification of “cultures” or “techno-complexes” of the Americas based on a distinctive set of lithic tool types, used to define the timing and widespread dispersal of Paleoindian populations [49]. Given the significance of fluting technology in the interpretation of the cultural history of the Americas, it is perhaps unsurprising that facile theories have come under intense scrutiny, and as described above, a range of theories have been advanced as to why the flute was made [2,8,42]. It is clear, however, that fluting was intentional and this point has been clearly demonstrated (e.g. [2,3]). A range of archaeological and experimental studies have demonstrated that the flute may be obtained by the application of several detachment techniques, including direct percussion, indirect percussion and pressure ([2,3,50,51]. By observing knapping traits (e.g., scar patterns, platform morphologies, ventral characteristics of channel flakes), it has been possible to differentiate the tools that were used for manufacture, including billets of organic (e.g., wood) or animal (e.g., bone, antler, ivory) materials; hammers made of soft to hard stone; a punch generally of an organic material; pressure flakers of organic materials for free-hand pressure; and a crutch of organic materials for chest or shoulder pressure. Most of these detachment techniques have been widely tested and observed in stone tool experiments and replications.

In the last few decades, the definition of distinct variations in fluting across North American Paleoindian technologies has been debated. Some researchers have made a distinction between “end-thinning” and fluting *per se* [33,52], also emphasized by the use of terms such as “technological fluting” and “morphological fluting” [53]. The process of end-thinning can occur at any phase during the *chaine opératoire* of the bifacial fluted point manufacture. End-thinning sometimes only results in the thinning of a preform, or preparing a hafting zone on a finished point. For instance, Clovis points may be end-thinned throughout the production process [33,54,55,56]: fluting was not always one of the last steps of point production as many Clovis point bases are constructed around prior end-thinning scars from earlier reduction stages. This differentiation between end-thinning and fluting means longitudinal flaking was already a well-established thinning technique, which shows a long-term history of the fluting concept that did not suddenly appear for symbolic or prestigious reasons, but rather might have evolved from this longitudinal flaking tradition [57,58]. This observation also applies to another sub-type, Dalton points, that are end-thinned throughout the bifacial production process. There is debate about whether Dalton points should even be considered morphologically “fluted” or “basally thinned”. The distinction concerns whether the end-thinning scars travel beyond the hafted area of the point [53].

**Fig 3:**
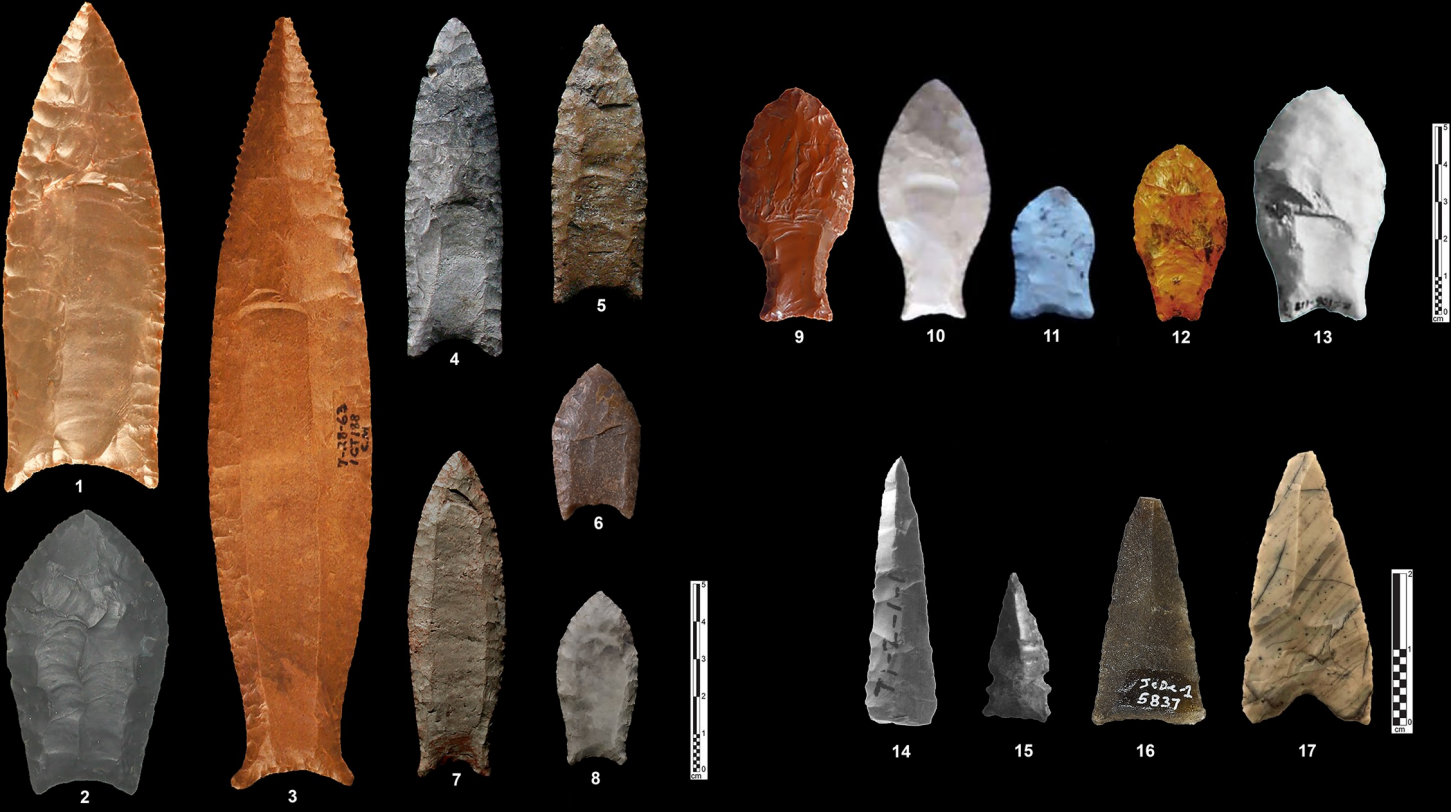
Various types of North and South American fluted points. 1–8: base-fluted points from North America– 1: Clovis point (Logan, Kentucky, modified from [60]:Fig 2); 2: Crowfield point (Addison, Vermont, modified from [60]:Fig 2); 3: Cumberland point (Colbert, Alabama, modified from [60]:Fig 2); 4,5: Gainey (Michigan, photo credits: T. Bennett Michigan Archaeological Society, http://micharch.org/wp/about-us/projectile-point-typology-and-dating/); 6: Folsom (Roosevelt, New Mexico, modified from [60]:Fig 2); 7,8: Barnes (Michigan, photo credits: T. Bennett Michigan Archaeological Society, http://micharch.org/wp/about-us/projectile-point-typology-and-dating/). 9–13: base-fluted fishtail points from South America– 9: Uruguay (modified from [85]: Fig 1); 10,11: Paso Centurión (Uruguay, modified from [86]: Fig 3); 12: Buena Vista (Uruguay, modified from [86]: Fig 4); 13: Siraba (Venezuela, modified from [80]: Fig 2). 14–17: tip-fluted points from northern America– 14,15: Site KkHh-3 (Canada, modified from [84]: Fig 7,8); 16: Site JcDe-1 (Canada, photo credits: Cité d’archéologie et d’histoire de Montréal, A. Vandal 2016—Creative Commons 4.0 (by-nc-nd), http://www.patrimoine-culturel.gouv.qc.ca/rpcq/detail.do?methode=consulter&id=204466&type=bien#.Xp9YOmgzbb0); 17: Saint-Pierre et Miquelon (photo credits: with the kind authorization from Projet Saint-Pierre et Miquelon 2019).

Though fluting characteristics have been used to relate stone tool types in North America, there is a considerable variation in the morphology of point types (e.g. [45,59–61]). It is clear that the fluting method in North America leads to the production of particular base-fluted point types (Fig 3: 1–8; see [62]: Fig 9.4 for a typo-chronology of the north American fluted points), the most famous of which are Clovis and Folsom points [32,33,63]. However, there are also a wide variety of sub-types from eponymous sites in the United States and Canada [42,44,64] such as, for example, Barnes [65], Colby [66], Crowfield [67], Cumberland [68], Dalton [69], Debert [70], Gainey [71], Goshen [72], Hazel [73], Northumberland [74], and Redstone [75] points.

In Central and South America, there are examples of so-called “fishtail” points (Fig 3: 9–13), which are base-fluted along a wide tang [76,77]. Significantly, only a minority of the fishtail points show fluting, and are mostly unifacial in appearance [78]. In Central America, examples are known from Mexico to Panama, and are dated from relatively few stratified sites, as for example, Los Grifos (Chiapas), Los Tapiales (Guatemala), and Cueva de los Vampiros (Panama) [79] and various places in Venezuela such as the El Jobo site [80]. Typical Clovis-like fluted points occur north of Costa Rica and Panama [76]. In South America, several sites are of the same age as Clovis sites in North America, such as Cerro Tres Tetas, Cueva Casa del Minero and Piedra Museo in Argentina, Quebrada Jaguay in Peru, and Fell’s Cave and Quebrada Santa Julia in Chile [46,81,82]. Made on stemmed points and not on lanceolate forms, South American fluted points differ in shape from their northern counterparts.

Other points are fluted from the distal part of the projectile (Fig 3: 14–17) and are usually found in the northern part of the American continent, although basal-fluted point exist in glaciated northeastern North America [83]. The points appear among Canadian Middle Dorset collections and less frequently in Early Dorset ones in the Late Paleo-Eskimo period (2500–1250 BP), such as numerous sites on various islands like for instance Killiniq, Southampton or Victoria. These “tip-fluted points” were produced by pressure with microblade-like spalls from the apex of a specialized blank; the method of fluting was applied at different stages of point manufacture, from the blank, to the preform, to used and broken points [4,84].

In North America, the fluting method has been viewed as part of a coherent technological tradition which spread rapidly with the very first hunters [45,46,87,88]. Comparisons of fluted points from northern and southern parts of the New World have influenced the debate on South America’s peopling and cultural origins [89,90]. Dating of fluted point assemblages in South America indicate that ages are as early as North American examples [76,77,91,92], raising a question as to how this distinctive knapping method appeared almost simultaneously in both parts of two continents. Difficulties in interpreting the presence of fluting in South America have fueled disputes about its origins, whether from migrations or diffusions amongs populations, or from technological convergence [76,78,93]. Because fluted points and basally-thinned points from the Western Hemisphere are a shared cultural trait, building models of human migration between North and South America based on one material culture trait remains contentious to this day.

### Fluting in the Old World?

**Fig 4:**
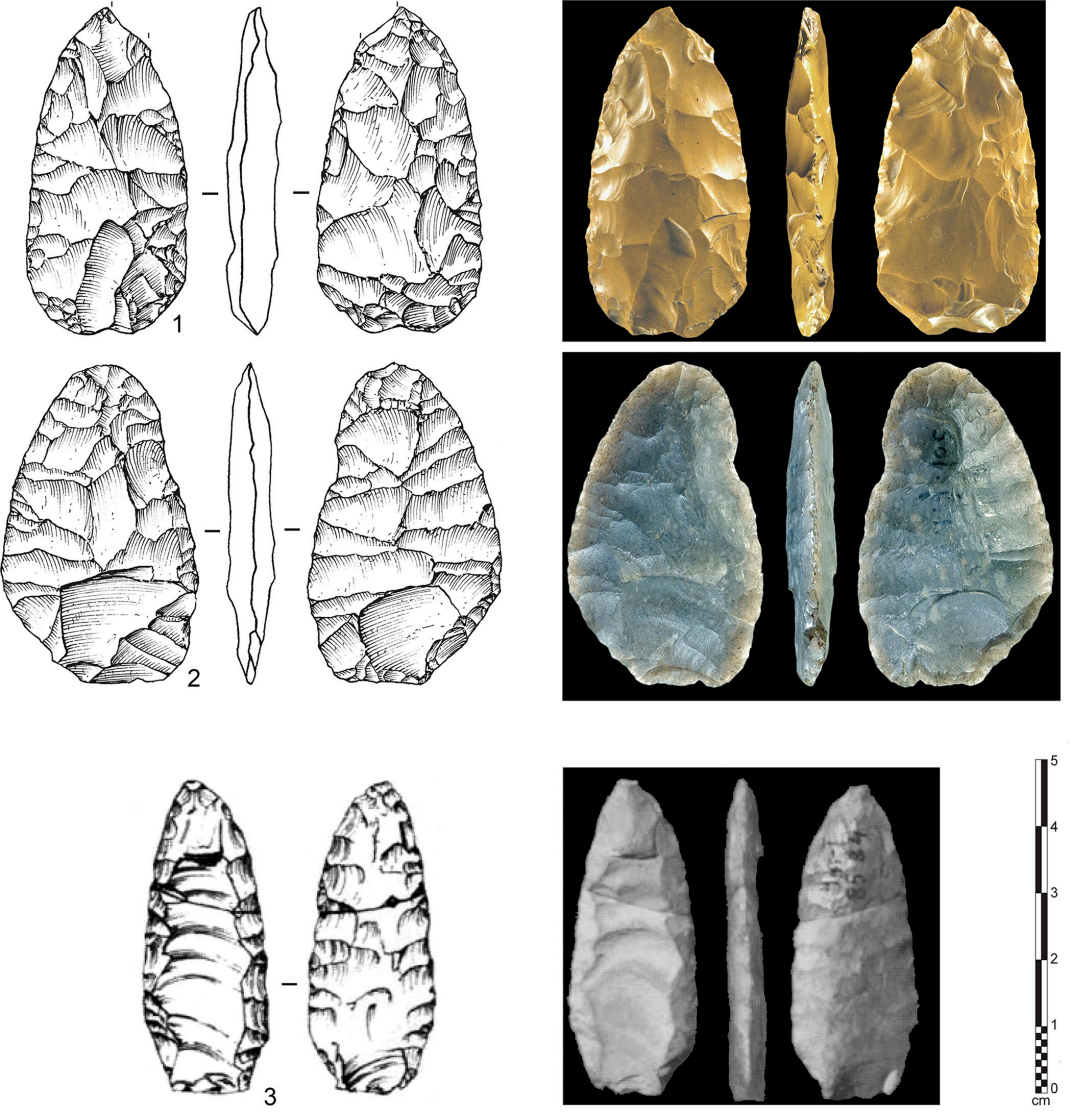
“Fluted points” from the Old World. 1,2: Laugerie Haute site, southwest France (photography used with the kind authorization from Musée National de Préhistoire, Les Eyzies); 3: Uptar site, Siberia (modified from [94]).

Although fluted points are characteristic of the Paleoindian record of the Americas, rare and isolated examples have been described from the Old World, particularly in Siberia and western Europe. Fluting occurs at the northeast Siberian site of Uptar [94,95] where a single-face fluted point was identified (Fig 4: 3). This thin bifacial piece is about 45 mm long and 18 mm wide and 6 mm in thickness. The flute scar is about 35 mm long and the point was found broken in two parts at the two thirds point of its total length. As a single, unique piece, it is unclear that it was part of a stone tool tradition. Furthermore, the fluted point comes from an insecure context without direct dating of the stratigraphic layer. Nevertheless, the artifact itself is a genuine and intentionally-fluted lanceolate projectile point with a clear flute scar obtained on a slightly convex surface. This potential techno-typological link between Siberia and North America is nevertheless not convincing enough, in the frame of the general specificities of material culture. The presence of such a tool in Siberia might rather illustrate assemblage variability which is sometimes neglected by traditional colonization models [94]. On the other hand, the Uptar point could represent the northwestern-most occurrence of North American fluted points, without implying an origin of fluting technology from Northeast Asia, where this knapping method is generally unknown.

Two bifacial pieces (Fig 4: 1,2) have been described as “potentially fluted” from Southwest France [96]. Discovered during the Denis and Elie Peyrony’s excavations in 1920 to 1925, the points come from the site of Laugerie Haute ([97]: Fig 142, No.11). Few details are known concerning the exact context of the discoveries, except that the bifaces were found in the western part of the site in a Middle Solutrean layer. In reporting these pieces, Smith [96] was not convinced about a link with fluted points of the Americas and reasonably attributed their formation to a non-intentional action during the shaping of the small foliated bifaces. Despite thus criticism, these pieces have been used as support for a possible European-North American connection [98], though this theory has been widely discounted (e.g. [99]). These Solutrean pieces are unusual in form, and our recent observations at the French National Museum of Prehistory at Les Eyzies support Smith’s original conclusions. One of the bifaces shows a narrow removal at the base of one face, being obviously part of the bifacial thinning of the piece. The second example shows two basal scars on each face, but the general aspect of the piece leads to interpret it as a small unfinished preform that was severely reshaped at its base, and most probably abandoned because of hinged removals. Thus, the shaping methods employed on these two bifaces are not comparable to an intentional fluting method. Furthermore, the absence of these types in the widely-investigated French Upper Paleolithic confirms a non-intentional process for these rare forms, and accordingly do not relate to a purposeful fluting method.

## The fluting method in South Arabia: From isolated discoveries to a new tradition in tool-making

### First discoveries in Arabia

**Fig 5:**
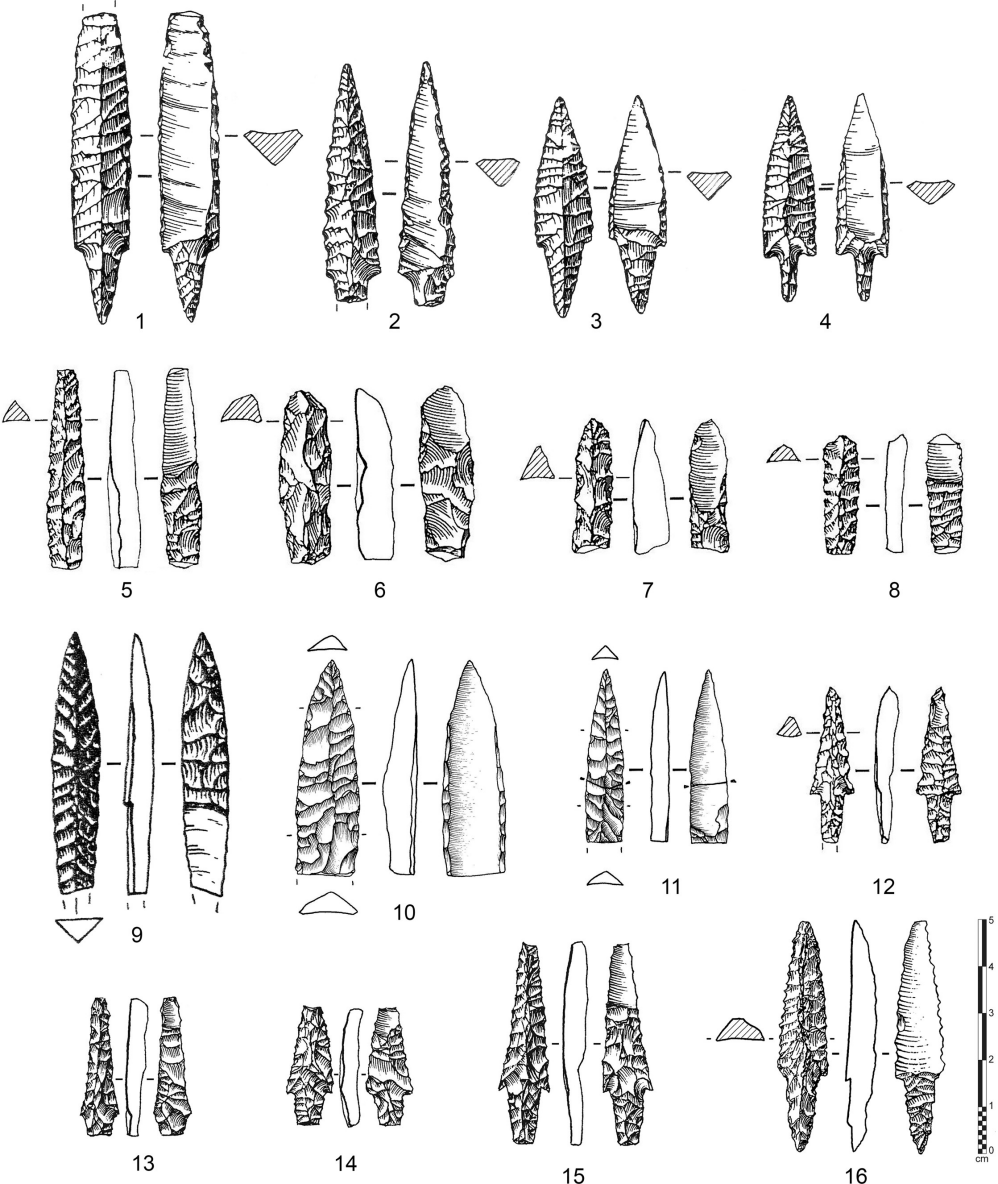
Various types of fluted points from Yemen and Oman. 1–4: Abr 1/3 site, Yemen ([112]: 143; [5]: 41); 5–8: Mahdi site, Yemen ([5]: 42); 9: Habarut site, Yemen ([101]: 227); 10,11: Gravel Bar Site, Yemen ([113]: 120); 12: Khor al-Hajar KHJ-1, Oman ([5,108]); 13–16: Suwayh SWY-1 site, Oman ([105]: 55).

The first mention of fluted pieces was from their discovery at Habarut in South Arabia in the early 1960s [100]. Habarut is now located in Yemen’s Mahra province, bordering the Dhofar region of Oman. The original investigators did not use the term “fluted points”, but provided examples in their illustrations (Fig 5: 9; see [101]). The longitudinal scars along the axis of trihedral points were interpreted as the effect of the excessive, and hard impact on hafted tools ([100]: 186).

Although fluting in Arabian assemblages were tentatively mentioned in the 1990s [101,102], the fluting method was first formally identified as an intentional knapping operation from surface sites in Oman and Yemen in the early 2000s [5,103,104]. The fluted points were referred to as fluted “trihedral points” as their cross-section was triangular (Fig 5: 1–4). The fluted trihedral points are now considered a sub-type of the “trihedral point tradition” that is widely spread over South Arabia. This lithic tradition is only observed in a restricted region, and therefore interpreted as an independent regional cultural development [6,105,106,107]. Evidence for this interpretation comes from a channel flake found on the surface site of Abr-1/3 in central Yemen and at Mahdi, in southeast Yemen, where a number of fluted trihedral projectile points were identified [5] (Fig 5: 5–8). The projectile points were found to be tip-fluted, illustrating the clear application of the fluting method (not impact damage). Other points occasionally showed proper impact fractures rather than genuine fluting, such as at the Oman Sea coastal site of Khor al-Hajar KHJ-1 [108] (Fig 5: 12). Further to the south, along the Arabian Sea coast, at Suwayh SWY-1, fluted points were identified in stratified context, and dated to ca. 7300 cal. BP [106] (Fig 5: 13–16). Other sub-types of trihedral fluted points were observed from surface and stratified sites in the inland plateau of Dhofar in south Oman [103,109] (fluted points and channel flakes are present) and in the Rub’ al-Khali Desert at the borders of Yemen, Oman and Saudi Arabia, at sites around Maitan [110].

Given that basal fluting was absent in southern Arabia at the time, a question was raised about the functional objective of fluting, which was not equivalent to “classic” American fluted points. The situation changed with the discovery of basally-fluted points, including even bi-fluted points, at the Manayzah rockshelter site in Yemen [111]. The Manayzah discovery raised questions about the functional aim of variably fluted forms and their knapping operations.

In the following sections, we describe the fluting occurrences from Manayzah, as this site is dated and has a complete *chaine opératoire* for fluting (composed of 6 fluted pieces and 21 channel flakes). In addition, the fluted assemblage from a newly discovered site, Ad-Dahariz 2, will be discussed, as it is an exceptionally rich surface site where 46 fluted points and preforms were found, as well as 11 channel flakes (S1 and S2 Tables). Ad-Dahariz 2 serves here as a reference for lithic experiments to identify bifacial thinning and detachment techniques used on Arabian fluted points, thereby allowing a deeper exploration about the function of fluting in Arabia. All necessary permits for the fieldwork and analyses were obtained from the General Organization for Antiquities and Museums in Yemen and the Ministry of Heritage and Culture in Oman.

### Fluted points from Manayzah (Yemen)

#### Dating fluted points at Manayzah

Discovered in 2004 in the Wadi Sana valley of south-central Yemen, the Manayzah rockshelter is an important site in understanding Neolithic occupation in South Arabia [6,111,113–115]. Manayzah is a stratified site, a rare occurrence for the region. Fluted technology is present in surface contexts as well as from excavated and securely dated contexts through a 2.20 m deep stratigraphy. The lithic assemblage, mostly made of local fine-grained chert, totals to 5063 excavated artifacts from a 15 m^2^ area and 2462 surface artifacts over an area of 158 m^2^. The lithic artifacts are composed of diverse bifacial products, with a high proportion of bifacial thinning flakes and various thin bifacial tools at different stages of reduction. Flake and irregular bladelet production from small multiplatform cores is also observed, including the use of nonlocal obsidian, which may have an origin ~400 to 500 km in distance. Varied types of arrowheads (n = 33) were found in association with the fluted items.

**Fig 6:**
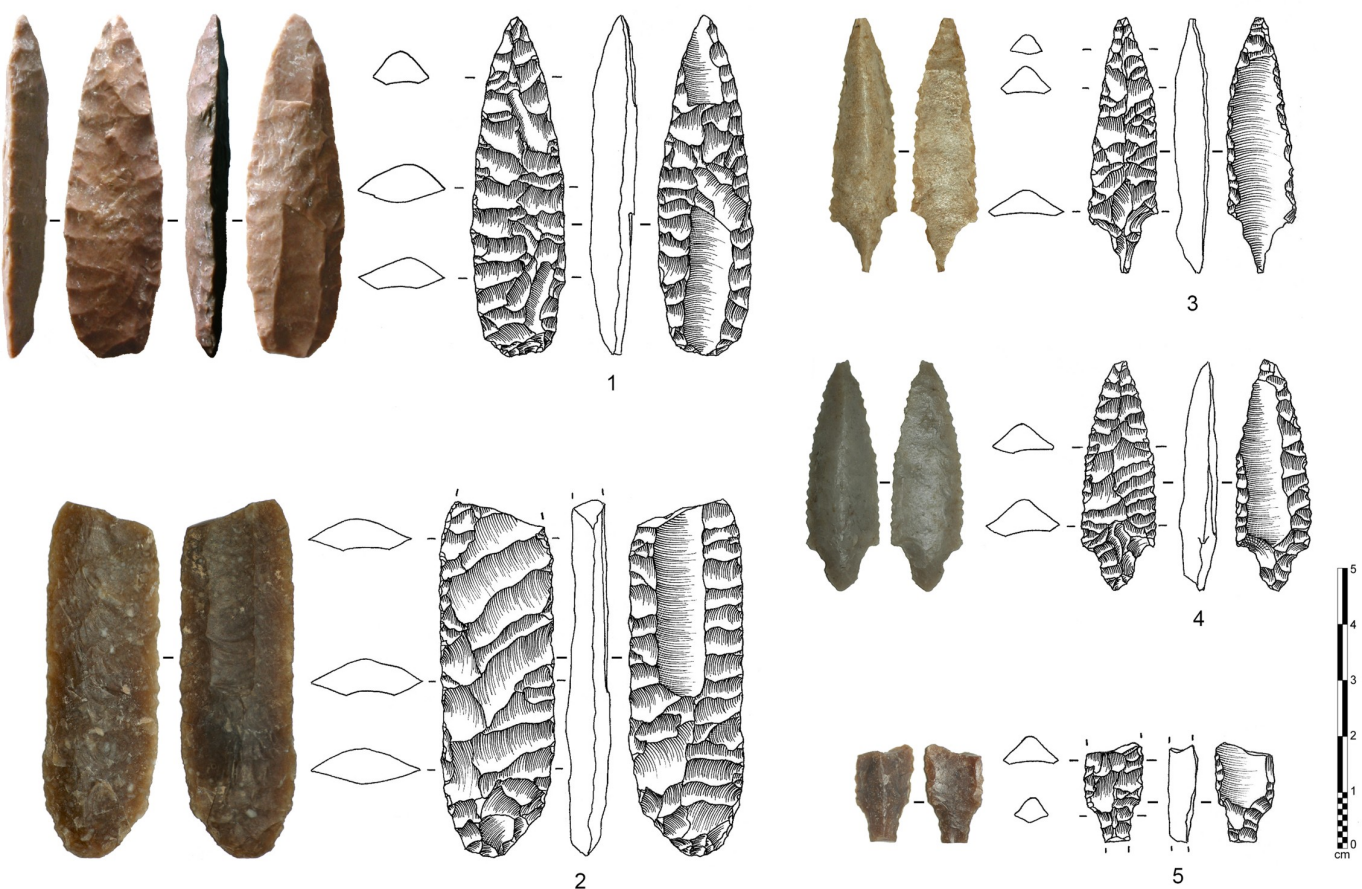
Various types of fluted points and preforms from Manayzah, Yemen. 1: double-fluted preform on a plano-convex elongated bifacial piece; 2: broken fluted preform on a bifacial elongated piece with symmetrical section; 3: base-fluted projectile point; 4,5: tip-fluted projectile points.

**Fig 7:**
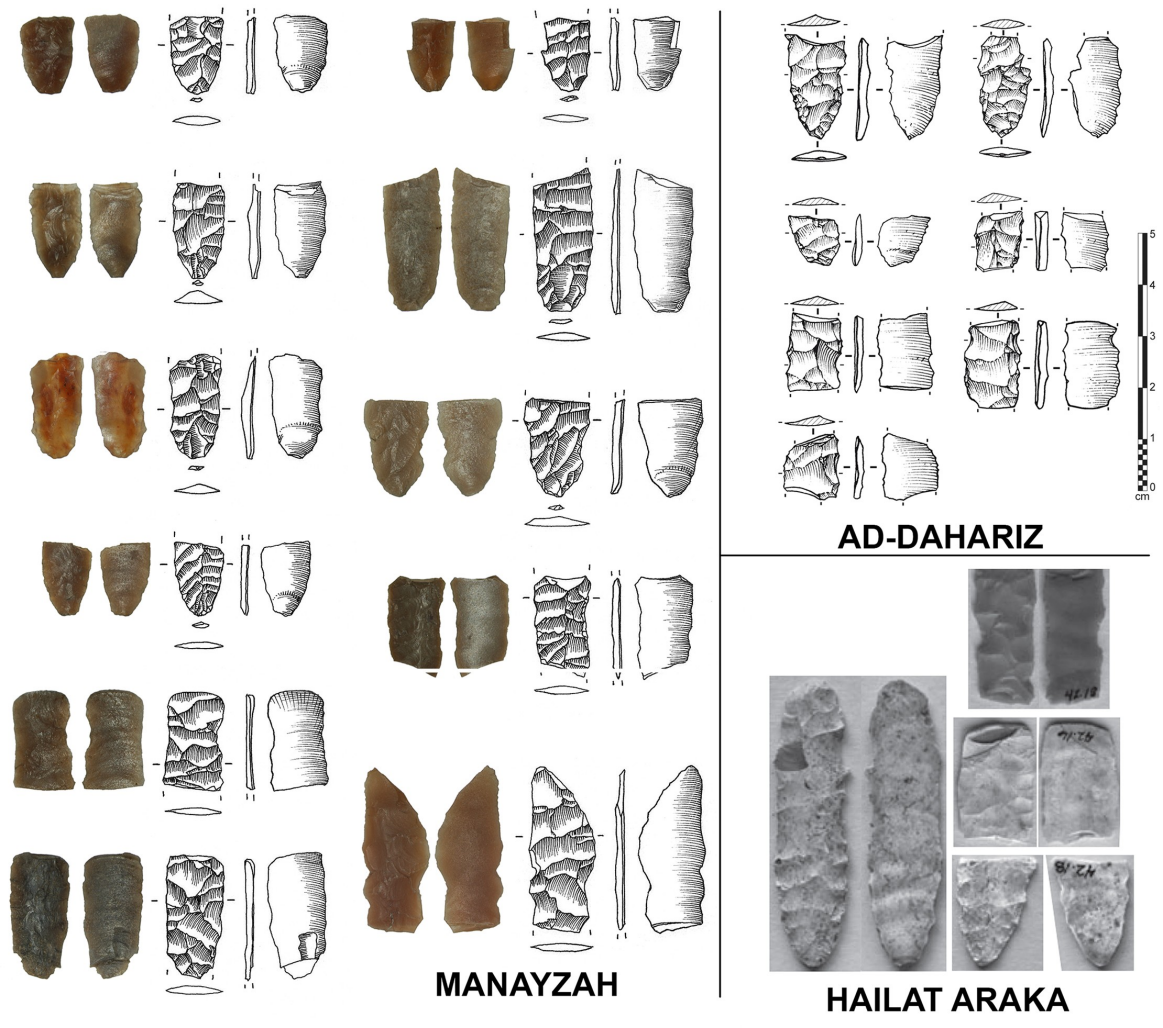
Channel flakes from southern Arabia. Left: Manayzah; top right: Ad-Dahariz 2; bottom right: Hailat Araka [109].

The fluting method at Manayzah is represented by complete and fragmentary fluted points (n = 3; Fig 6: 3–5), fluted preforms (n = 3; Fig 6: 1–2) and channel flake fragments (n = 21; 19 from stratified contexts and 2 from surface; Fig 7). The fragmentary channel flakes are proximal, medial and distal pieces and do not conjoin, representing at least 21 individual fluting operations. The presence of channel flakes confirms that fluting was completed on site. The fluted elements and channel flakes appear in several stratigraphic horizons, radiocarbon dated to 7133 ± 51 BP (6086–5896 cal. BC) and 6902 ± 41 BP (5885–5716 cal. BC). Fluted pieces at Manayzah therefore date to ca. 8000–7700 cal. BP. There are no older examples of fluting in the deeper excavated layers at Manayzah.

Manayzah places fluting in South Arabia in a clear chronological framework, situating it in the South Arabian Neolithic [7,109,116]. This regional Neolithic tradition does not appear to have cultural links with neighboring Neolithic cultures. This is based upon divergences of contemporary projectile types in southern, central and northern Saudi Arabia and the presence of Pre-Pottery Neolithic forms in the Levantine region of the Fertile Crescent [117–119]. A set of other associated typo-technological tool classes are found in the Neolithic period in South Arabia. Closely related to the fluted points, arrowhead types include the unfluted trihedral points [105–106]; at least three different types are observed at Manayzah, having a highly regular triangular section, as observed on most of Arabian fluted points [110,113]. The unfluted trihedral points appear to be contemporaneous, and older than the fluted forms, dating to ca. 8200/7500 cal. BP in Yemen.

Trihedral point types, including fluted trihedral points (Fig 5: 10–11), have been identified at surface assemblages from several sites around Manayzah, particularly at Wadi Sana Gravel Bar Site (GBS) and also in the stratified site of Khuzmum 045-1A [113,115,120,121].

#### *Chaine opératoire* of fluting from Manayzah

**Fig 8:**
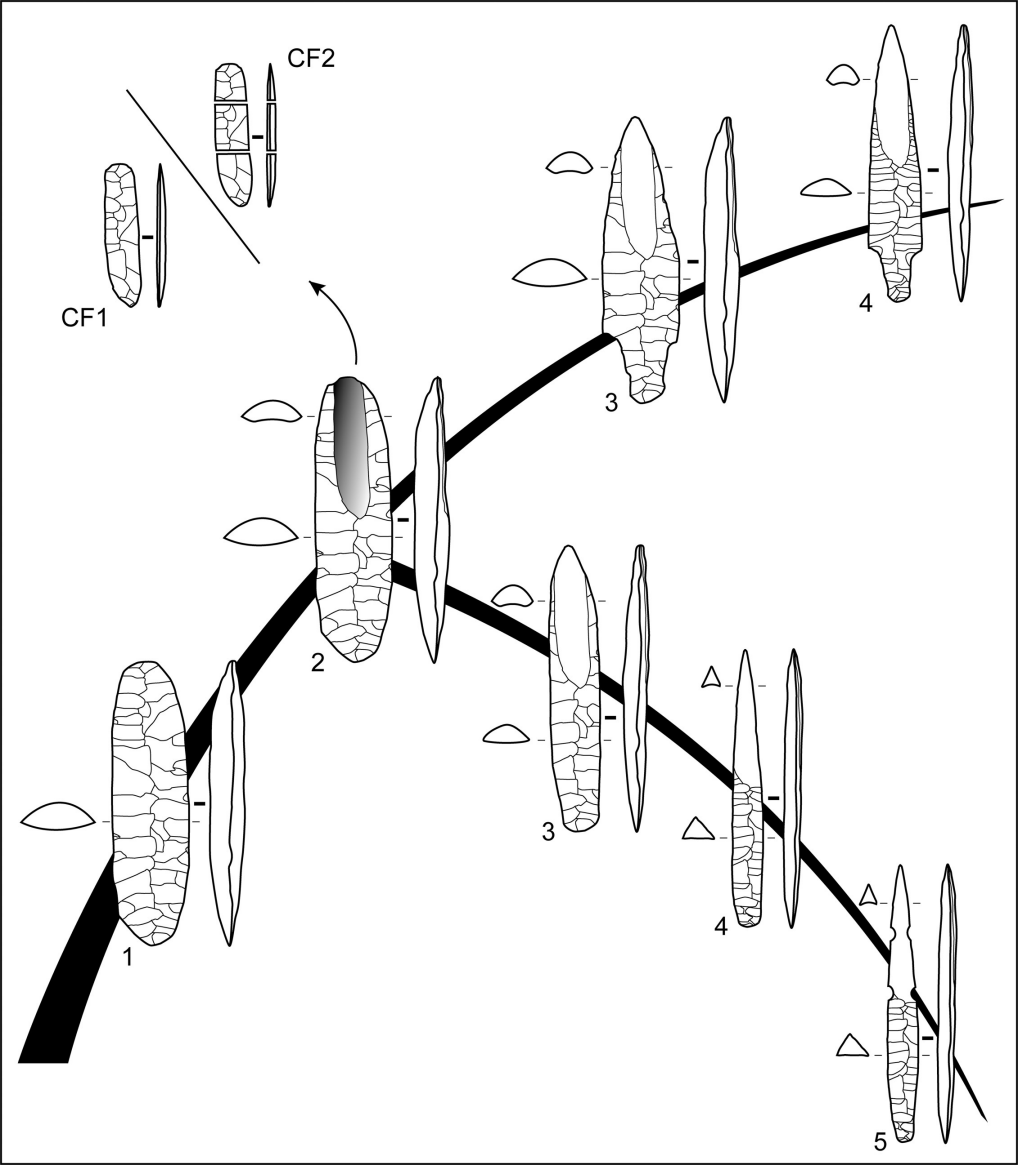
Theoretical *chaines opératoires* of fluted projectile points from southern Arabia. 1: bifacial preform, 2: fluting along one face, producing a complete (CF1) or broken (CF2) channel flake, 3 to 5: progressive reduction sequence of the final fluted point (pressure shaping/*façonnage*). The upper point is of a Manayzah type with a shouldered tang, and the lower one is of a Ad-Dahariz type with lateral notches.

The stone tool assemblage at Manayzah, consisting of all stages of the reduction sequence, provided an opportunity to examine the *chaine opératoire* for fluted points (Fig 8). Three principal stages were identified in fluted point manufacture: 1) the selection of raw material and the pre-shaping of a bifacial piece, 2) the fluting process itself, and 3) the shaping of the final projectile form, typically having a triangular cross-section. Each main stage of reduction is summarized below.

Stage 1: raw materials were procured from a range of local outcrops, including sources near bedrock exposures and in secondary river contexts. Cobbles and pebbles from wadi beds were primarily selected and widely-used at Manayzah. The river clasts consisted of different materials, the majority of which were varieties of chert, but also included chalcedonies and jaspers. Because of their high-quality attributes for knapping, cherts were favored, especially for their ease in pressure-flaking, although some of the chert was improved using heat treatment. A second source for raw material was atop the nearby Hadramawt plateaus, just above Manayzah, where high quality chert from thin tabular blocks was abundantly available across large outcropping zones [113]. Fragments of such blocks were recovered in excavations. Although present throughout the stratigraphic sequence, obsidian was not used for fluted points production. After selecting good quality raw materials, knappers typically shaped small and elongated bifaces with a plano-convex to an asymmetrical bi-convex cross-section, either from a flat chert block or a thick flake (Fig 8: 1). The percussion tools used in bifacial thinning were hard organic and/or soft mineral materials (as suggested at the archaeological sites by the lipped platforms, general characteristics of bifacial thinning flakes, and the flat and long scars on the preforms and the final products), prior to applying pressure flaking for finalizing the preform. Just before fluting, the bifacial preform was not necessarily pointed, as this was accomplished during the last stage of the final retouching of the fluted point.

Stage 2: fluting was made along the longitudinal axis from one extremity of the bifacial piece, typically on the ventral face, which is the least convex face (Fig 8: 2). The knapper frequently applied intensive abrasion to the striking platform to avoid crushing the channel flake’s platform under the action of the pressure- or percussion-tool. Channel flake platforms were usually punctiform or linear, and bulbs were usually small and restrained, and clearly delimited on the proximal ventral face. The obtained channel flake was flat, linear and regular in profile. Knapping showed some differences in the fluting process, with planed fluting originating at the tip, the base or both extremities of the bifacial preforms. This variability may be a product of differences in knapper’s objectives, which may range from functional advantages of fluting, technological attempts to create the longest flute possible.

Stage 3: when fluting was successful, or at least satisfying to the knapper, the fluted biface was thinned by bi-lateral retouch to obtain a strictly triangular cross-section (trihedral) or a plano-convex cross-section with a triangular tendency (Fig 8: 3–5). The retouch was made from the fluted face only, the latter not being reworked, or only very slightly, after the flute was produced. In shaping a pointed form at the tip (distal end), the knapper applied less thinning at the base (proximal end), which is represented as either a thin tang or shouldered with contiguous lateral edges of the arrowhead.

One fluted bifacial preform recovered on the surface of Manayzah shows two opposed fluting removals from the same face (Fig 6: 1). On a small almond-shaped biface with an asymmetric, biconvex cross-section, bifacial thinning was accomplished by semi-abrupt pressure-flaking retouch on the obverse, and by low angle retouch on the reverse. One first fluting attempt failed, as seen from the scar of a hinged channel flake. A second fluting attempt, also hinged, from the opposed extremity of the bifacial piece probably shows the knapper’s final goal was to have a complete flute along the full length of the piece. As this small bi-fluted biface was not retouched after the fluting extraction, it is considered to be an abandoned preform rather than a finished tool. The reason as to why the fluting scar should have been placed along the length of the fluted piece is intriguing. If there was a functional reason, the fluting scar should have a role to play either in tool performance, or in its thinning process, just before it was considered as a finished projectile point. Otherwise, a cultural reason may be considered, as a complete flute would be the desired outcome.

### Fluted points from Ad-Dahariz 2, Oman

#### The Ad-Dahariz 2 site and its lithic assemblage

Fluted points and channel flakes have recently been discovered in southern Oman, at Khor Ad-Dahariz, one of the principal lagoons of the Dhofar region and part of the plain of Salalah. A paleo-mangrove has been detected along with several archaeological sites. Located at 1.5 km from the sea, the Neolithic site of Ad-Dahariz 2 covers more than 3.5 hectares and is today located on a fossil beach, on a slight promontory overlooking the lagoon. Ad-Dahariz 2 is one of the rare Neolithic coastal sites of Dhofar, and has now provided evidence of occupation in the plain of Salalah, estimated to date to the Oman Middle Neolithic (ca. 8500–6500 cal. BP) [106]. A 1 x 1 m test-pit had a thin stratigraphy (~30 cm in depth), with Neolithic artifacts found extending down to 10 cm from the surface. Artifacts from surface were selectively collected (retouched tools and channel flakes), while the remainder of the uncollected pieces were observed and described on site by a team of five to six archaeologists who spent a cumulative 240 hours documenting the site surface.

**Fig 9:**
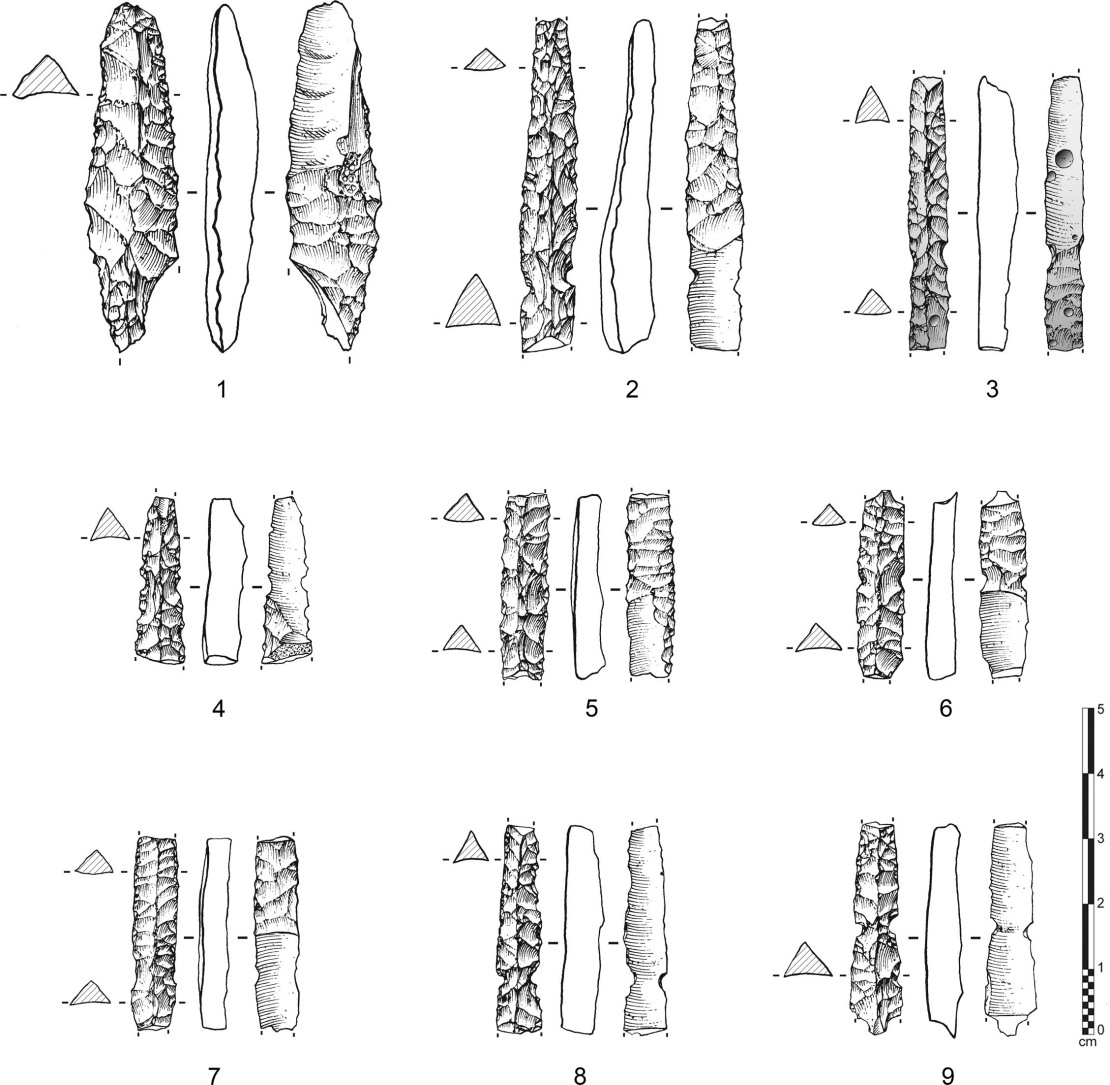
Various types of fluted points and preforms from Ad-Dahariz 2, Oman. 1: broken tip-fluted preform on a bifacial piece with a triangular section; 2–9: medial fragments of tapered fluted projectile points with triangular section (no. 3 is burnt).

The lithic assemblage from Ad-Dahariz 2, exclusively on very fine-grained chert, is characterized by numerous fragmentary and rarer complete trihedral points at different stages of production, with almost half of the points showing fluting removals (Fig 9). Among more than three hundred of collected lithics, a total of 39 fragmentary fluted points was found (36 from surface, 3 from the test-pit). In addition, seven fluted plano-convex bifacial pieces were abandoned preforms. In addition, 11 fragmentary channel flakes were identified (proximal and medial parts only; Fig 7). The fluted elements were associated with 48 unfluted trihedral points and 16 unfluted plano-convex bifacial pieces, with the presence of three thin and flat foliates and the same number of undiagnostic retouched blanks. The presence of the channel flakes indicates on-site fluting, although no refits could be made between channel flakes and fluted pieces.

#### Fluted and unfluted trihedral points: Morphology and metrics

The shaping and thinning (*façonnage*) of thin fluted and unfluted trihedral points at Ad-Dahariz 2 appears to be made by the application of fine pressure flaking. Trihedral points were systematically shaped in a bifacial manner. Only one or two discrete examples show trifacial shaping, with removal scars coming from the superior ridge, though trifacial thinning procedure was rare. In terms of typology, the points, whether fluted or unfluted, have two (n = 11 fluted; n = 4 unfluted) to four (n = 2 fluted) lateral notches, symmetrically located compared to the central axis of the piece. The notches were systematically produced using direct retouch, from the flat inferior face. Retouch appears as a notch on each edge of the non-flat face.

The type of fluted points from Ad-Dahariz 2 differs from the types recovered from eastern Yemen. The fluted points from south-western Oman are often tapered (narrower and longer) and with a very standardized and regular equilateral triangular section. As seen at Ad-Dahariz, the points can have lateral notches and no tang, while fluted points from Yemen usually have a small tang and no notch. These particularities at Ad-Dahariz 2 are also present at Hailat Araka, another site in the Dhofar region [109]. Hailat Araka is located inland at some 150 km north of Khor Ad-Dahariz and the Indian Ocean littoral. This suggests a regional specificity in final point forms that can only be confirmed by additional site surveys in the future. The assemblage at Hailat Araka yielded 30 complete or fragmentary channel flakes. A total of 26 channel flakes were from a 50 cm deep stratified context, with a radiocarbon date of 7160 +/- 81 uncal. BP (see [109]: Figs 2 and 4), indicating an *in situ* fluting operation, as also observed at Manayzah and Ad-Dahariz.

At Ad-Dahariz 2, it is noteworthy to observe that the fragmentary fluted points are medial (n = 36) and distal-medial parts (n = 3). No complete fluted point has been found so far. Among the unfluted trihedral points, the same observation is made, as medial parts represent the highest proportion (n = 34), while distal-medial and basal-medial parts (n = 7) are as numerous as distal parts (n = 7). The breakage morphology on fluted and unfluted point fragments are predominantly with a ‘*languette*’ morphology (n = 73), then split (n = 16), and more rarely with a transverse diagonal morphology (n = 4).

**Fig 10:**
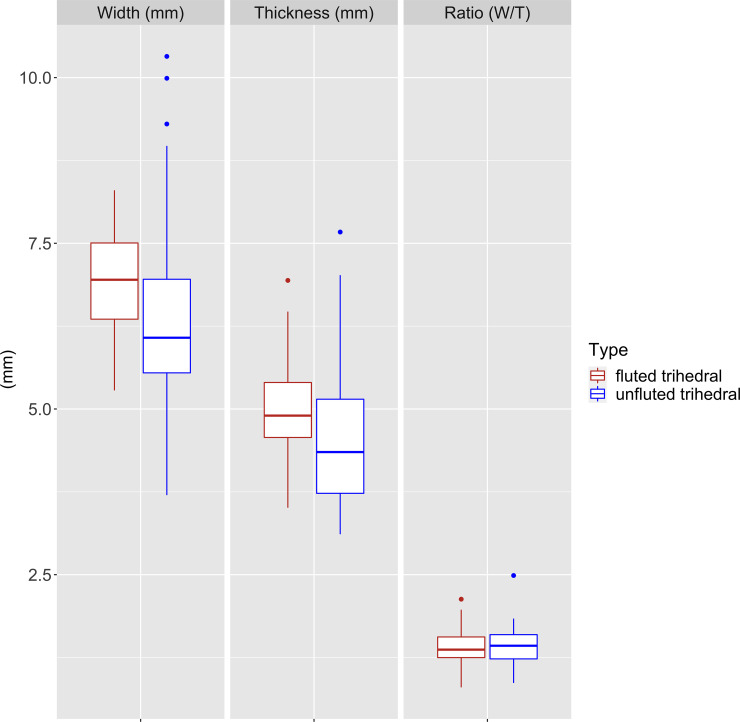
Boxplot diagrams, Ad-Dahariz 2. Comparisons between maximum width in mm (left), maximum thickness (center) and maximum width / maximum thickness ratios (right) between fluted and unfluted trihedral points.

**Fig 11:**
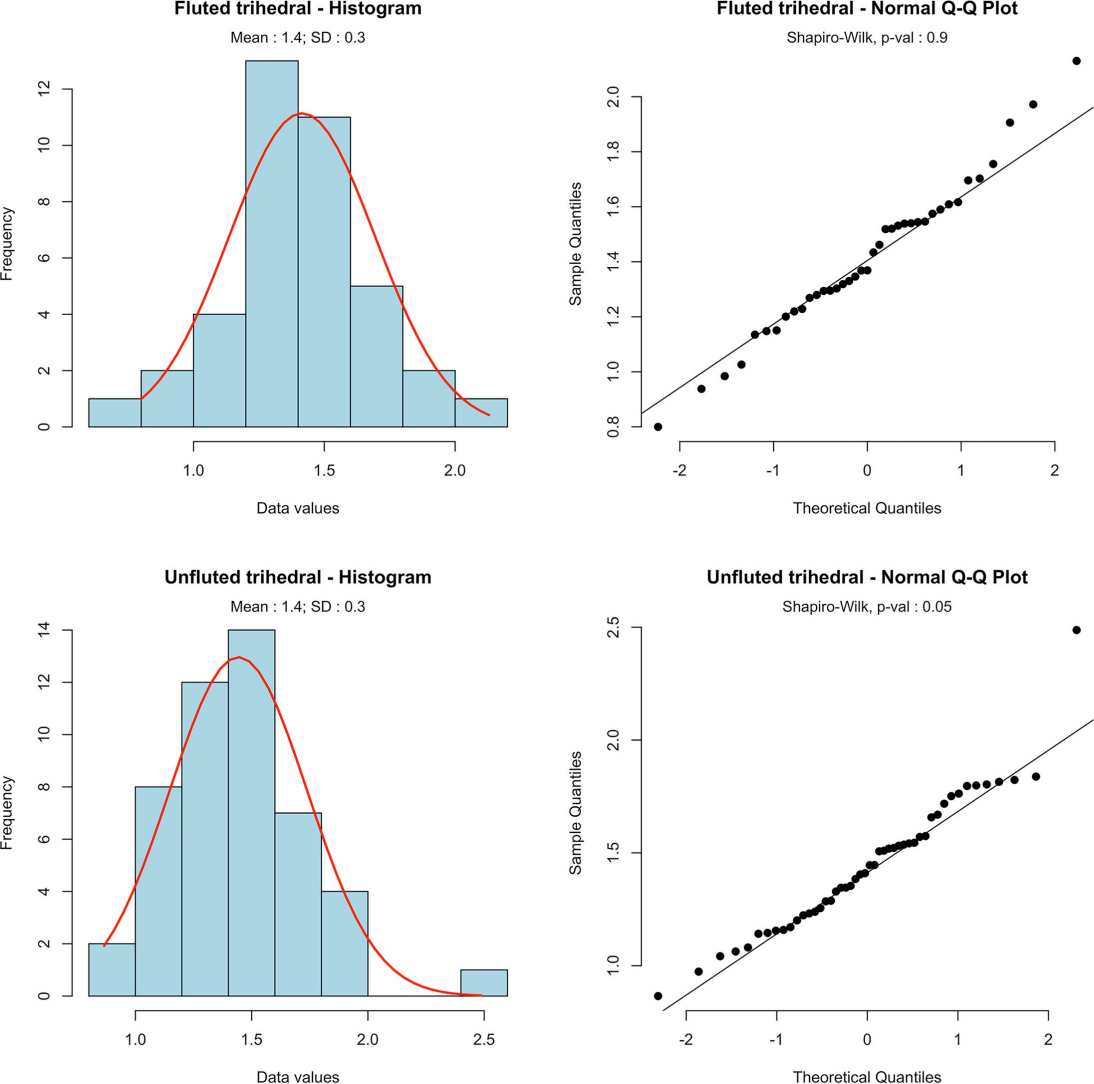
Distribution of the ratio of fluted and unfluted trihedral points from Ad-Dahariz 2. With kernel density estimation and Shapiro-Wilk test.

Considering the fragmentary nature of the projectile points, we first examined their width and thickness distribution and constructed an index based on the ratio of width divided by thickness. This ratio helped in assessing similarities and differences between unfluted and fluted materials. The boxplot diagrams (Fig 10) tend to confirm the similarity in the morphometrics of both groups. To investigate if there is a statistically significant difference between the means of the ratio of fluted and unfluted trihedral points, we performed a Student t-test (Fig 11). Frequency distributions, density estimation and Q-Q plots show that the ratio of fluted and unfluted trihedral points follow a normal distribution. This result is confirmed by Shapiro-Wilk normality tests (p-value(fluted) = 0.9, p-value(unfluted) = 0.05), and both tests have a p-value equal or greater than 0.05, which indicates a normal distribution of the data. The t-test does not show a significant difference between the ratio of fluted and unfluted trihedral points (t(85) = -0,447, p-value = 0.655).This result confirms the initial observation that there is no significant difference between the two types of trihedral points.

The quantitative data highlights similarity in the morphologies and sizes of both unfluted and fluted points, suggesting a lack of differentiation in the functional aspect of these pieces. The analysis attests to the presence of two groups that are both highly standardized, while de-valuing the functional discrimination of the fluted pieces. Trihedral projectiles were apparently not used in a different fashion, fluted or not. The aim of the fluting is therefore somewhat enigmatic, suggesting that social or cultural reasons could account for their co-occurrence with similar, unfluted material.

## Lithic experiments on Arabian fluting technology

Ad-Dahariz 2 yielded an adequate sample of both unfluted and fluted trihedral points, providing an opportunity to design and conduct lithic experiments on the fluting process observed at the site. The experiments were focused on the fluting method in particular, in an attempt to distinguish among several fluting modalities and detachment techniques.

### Experimental aims

The lithic experiments had three main goals: 1) to demonstrate the degree to which knappers intentionally fluted Arabian points (i.e., the type of knapping involvement, as revealed by choices in the detachment technique); 2) to better understand the relations between point technology and function (the range of detachment techniques used in the Neolithic of South Arabia was tested by comparing the experimental material with archaeological examples from the Manayzah and Ad-Dahariz 2 sites); and, 3) to estimate the degree to which cultural transmission accounts for the fluting at both Manayzah and Ad-Dahariz.

One of the first experimental procedures centered on addressing different fluting modalities to identify the *chaine opératoire* of fluted trihedral arrowheads. As the fluting method was spread among southern Arabian Neolithic societies, it seemed important to determine the degree to which manufacturing skills were involved in lithic production. Experiments on fluting and trihedral shaping (thinning, *façonnage*) also considered the functional aspect of those points. In the end, the lithic experiments proved worthwhile, providing relevant information about the skills involved in producing fluted and unfluted trihedral points, and here we review the main findings.

### Main morpho-technical characteristics of archaeological points from Manayzah and Ad-Dahariz

With respect to archaeological examples of unfluted and fluted points, four key observations were made: 1) heat-treatment is observed at both sites on some finished points and preforms; 2) finished points are triangular or sub-triangular in cross-section; 3) finished points are regularly and finely retouched by pressure from the two sides of the fluted or very flat ventral face; and, 4) finished points typically have mean dimensions around 60 to 70 mm length, 5.5 width and 5 mm thick. With regard to the fluting method itself, three main observations were made: 1) finished points had unifacial fluting that could be tip-fluted or base-fluted, and some unfluted points are similar in style and size (observed mostly at Ad-Dahariz); 2) channel flake platforms indicate frequent preparation of a slight “spur” bearing strong abrading; and, 3) the extension of fluting has a mean of ca. 50 mm in length (observed at Manayzah and other neighboring surface sites where complete fluted points have been discovered), 10 mm in maximum width and 1.8 to 2 mm in thickness.

### Experimental procedures

The main experimental objectives were to establish the degree of intentionality and control in the fluting process through the determination of knapping modalities, including detachment techniques. We followed three main stages of the *chaine opératoire* identified at Manayzah: 1a) selection of raw material (either a thick laminar flake or a small chert slab, see S3 Table for details on raw materials used in the experiments) and pre-shaping of an elongated bifacial piece with a plano-convex cross-section (using both direct percussion with soft stone and pressure using a bone-tipped tool); 1b) (optional) heat treatment in a fire pit; 2) preparation for fluting using pressure flaking with a bone-tipped tool, and application of the fluting process using different detachment techniques; and, 3) use of trihedral pressure shaping of arrowheads employing a sharp bone-tipped tool.

**Fig 12:**
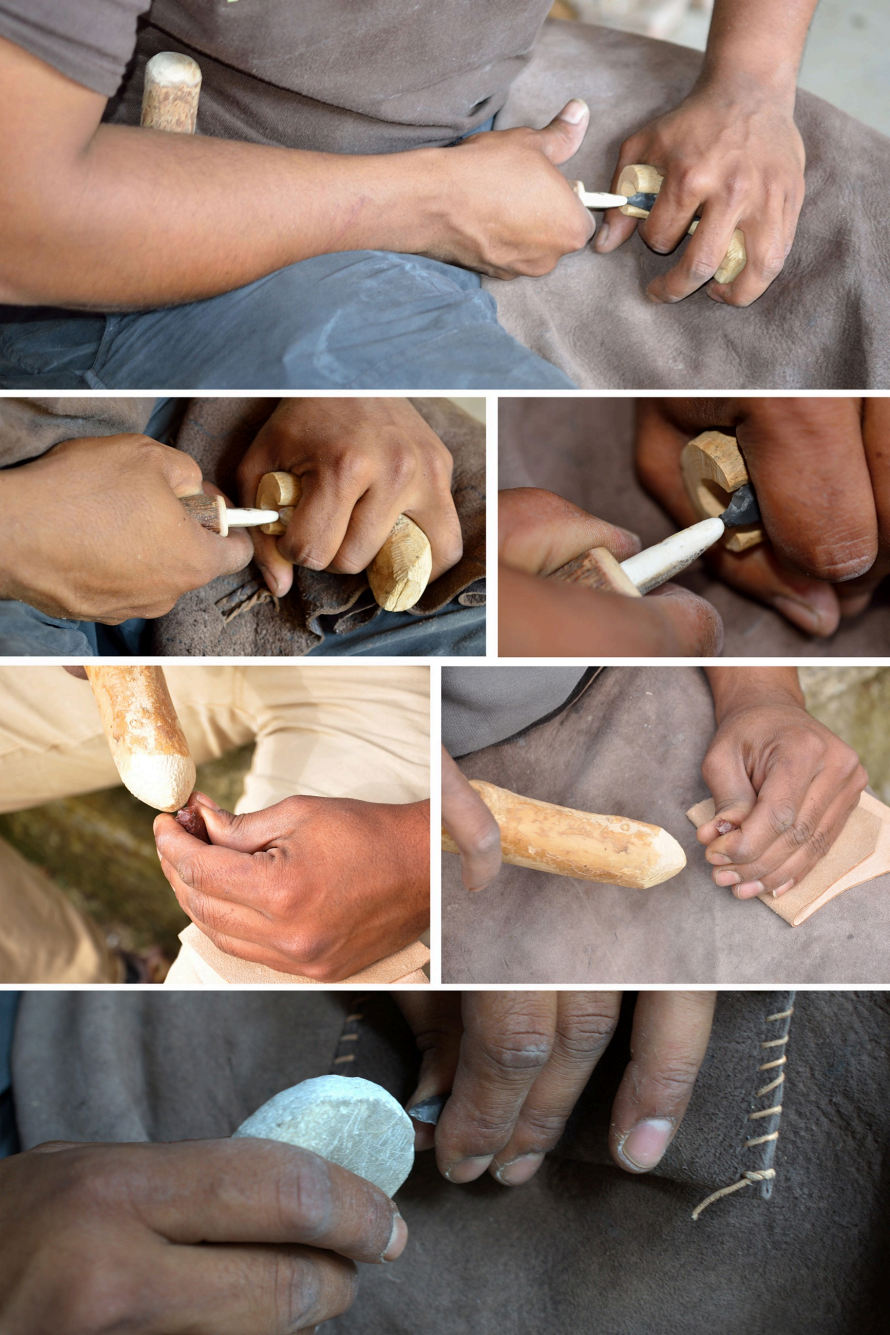
Examples of detachment techniques used during the knapping experiments. Pressure, percussion with hard wood and percussion with soft stone.

Experiments were conducted between 2013 and 2018 by a single knapper for consistency (JV, an individual who is a very highly-skilled knapper with more than twenty years of experience and who conducts stone tool knapping usually on a daily basis), using mainly heat-treated flint and chert and a tool-kit composed of hard wood, mammal bones and soft stone. Different fluting modalities were explored through use of a series of 160 pieces including multiple fluting tests (n = 226 fluting tests). Systematic tests were carried out for each potential detachment technique (Fig 12) using knapping tools of different weights (direct percussion using soft stone and hard woods, indirect percussion using antler, and pressure flaking for detachment with bone or antler) (Pelegrin’s mode 1b and 2 [122]).

### Experimental results

With a focus on fluting detachment techniques, the use of bifacial preforms was systematized (with plano-convex cross-sections). These were made from thin slabs or thick laminar flakes using soft stone to roughen the blank and to conduct pressure flaking to shape the anticipated fluting surface. The flaking platform was standardized using pressure flaking (lateral removals intended to create a slight spur) and abrasion. With respect to fluting modalities, 21 were conducted with pressure, 77 with soft-stone direct percussion, 47 with antler direct percussion, 58 with hard wood direct percussion and 23 with indirect percussion. We carried out numerous tests, including detachment techniques that had no observable similarity with the archaeological series. This was done for comparative purposes and in order to have representative and diagnostic elements (typical knapping accidents and specific morphologies of channel flakes and scars).

**Fig 13:**
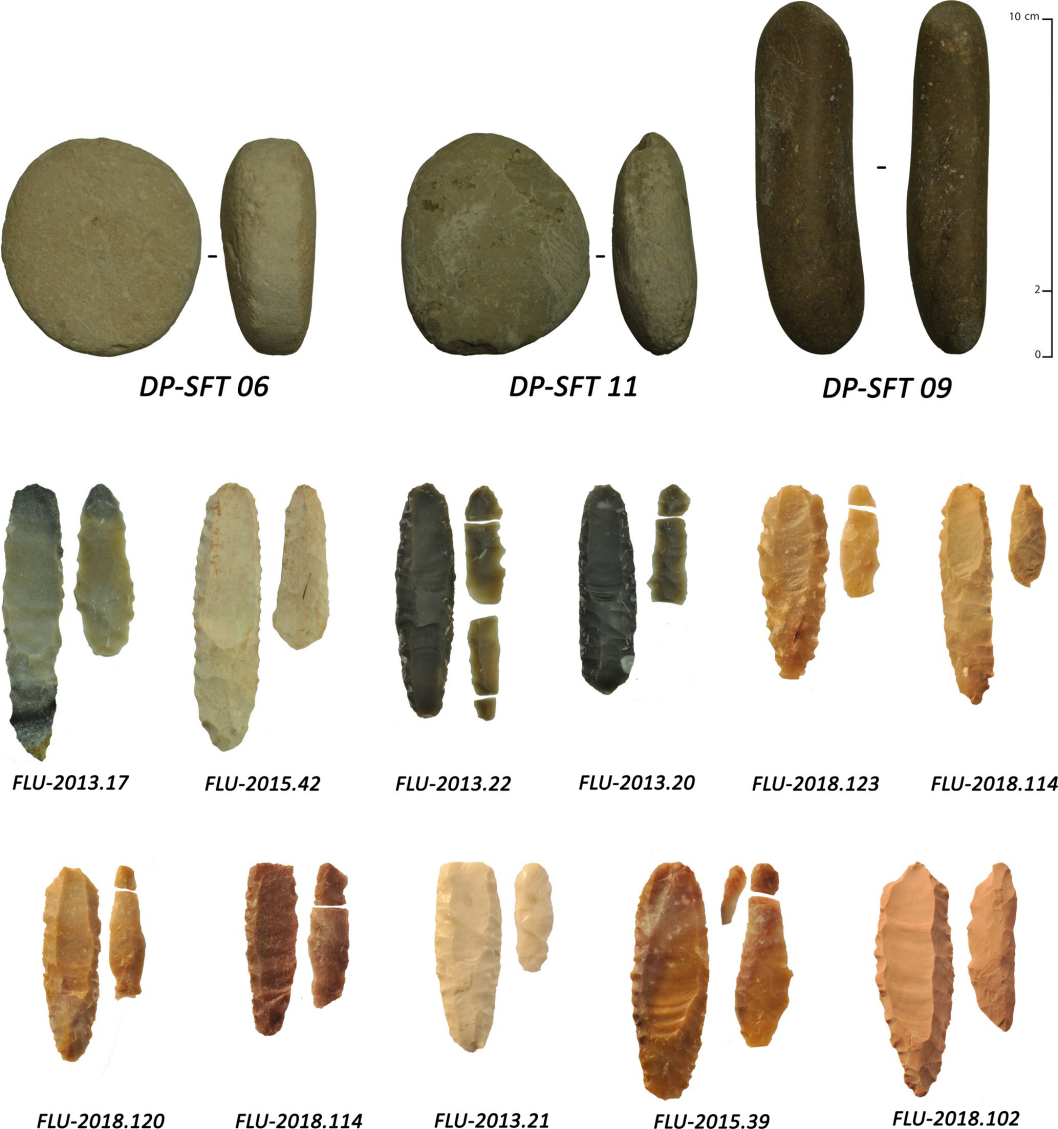
Key results of experimental fluting by direct percussion with soft stone. Top: fluted pieces are shown with their channel flake; bottom: some of the soft stone hammers used in the experiments.

For fluting with direct percussion, soft stones, antlers, and hard woods were used. Soft stone hammers were made of fine-grained limestone and sandstone (a few sandstone processing tools are present at Manayzah), weighing between 116 g and 245 g (mean weight of 162 g). Using an adequate amount of strength, this detachment technique allowed for an attainable fluting length, while minimizing knapping accidents such as hinge or plunging fractures. Channel flakes were regular in thickness, with sub-parallel edges. Breakage of the channel flakes was not systematic, but when broken, three fragments were frequently observed, with medial fragments being the largest in size. The distal ends were generally a little wider and thicker than the body of the channel flake, sometimes ‘vibrated’ or slightly hinged. The platforms were faceted and narrow, sometimes overflowed, and the bulbs were in high position and pronounced. The results were consistent with the archaeological assemblages, but limited in extent with respect to fluting length (mean of 26 mm) and overly thick distal ends. This led to a repetition of tests to improve results, though without notable success (Fig 13).

**Fig 14:**
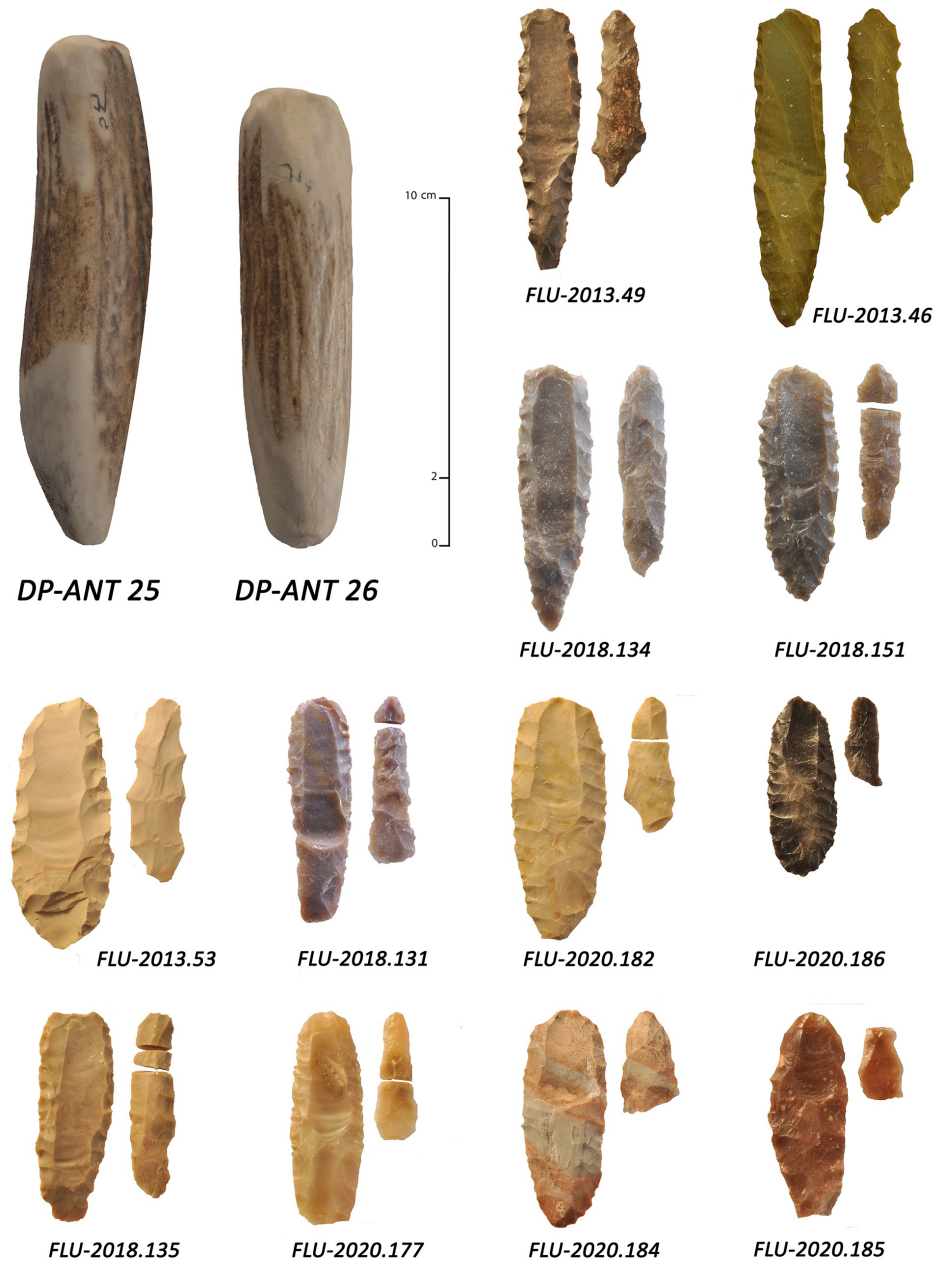
Key results of experimental fluting by direct percussion with antler. Top: fluted pieces are shown with their channel flake; bottom: some of the antlers used in the experiments.

Antler hammers were made of Virginia deer (*Odocoileus virginianus*), red deer (*Cervus elaphus*) and moose (*Alces alces*), weighing between 66 g to 262 g (mean weight of 145 g). The strength was difficult to balance with this detachment technique, but the use of the lightest tools limited accidents. However, numerous hinge fractures and limited plunging were noted. Channel flakes were thin (2.7 mm on average), with globally large platforms, slightly pronounced bulbs, marked lips and generally slight undulations. Breakages were frequent and the parallelism of the ridges was frequently more regular than with soft stone but a little less than with hard wood. The extent of fluting was close to what was observed with hard wood (38 mm) but the frequent overly-thick distal ends were once again a recurrent problem and difficult to overcome, even with repeated tests (Fig 14).

**Fig 15:**
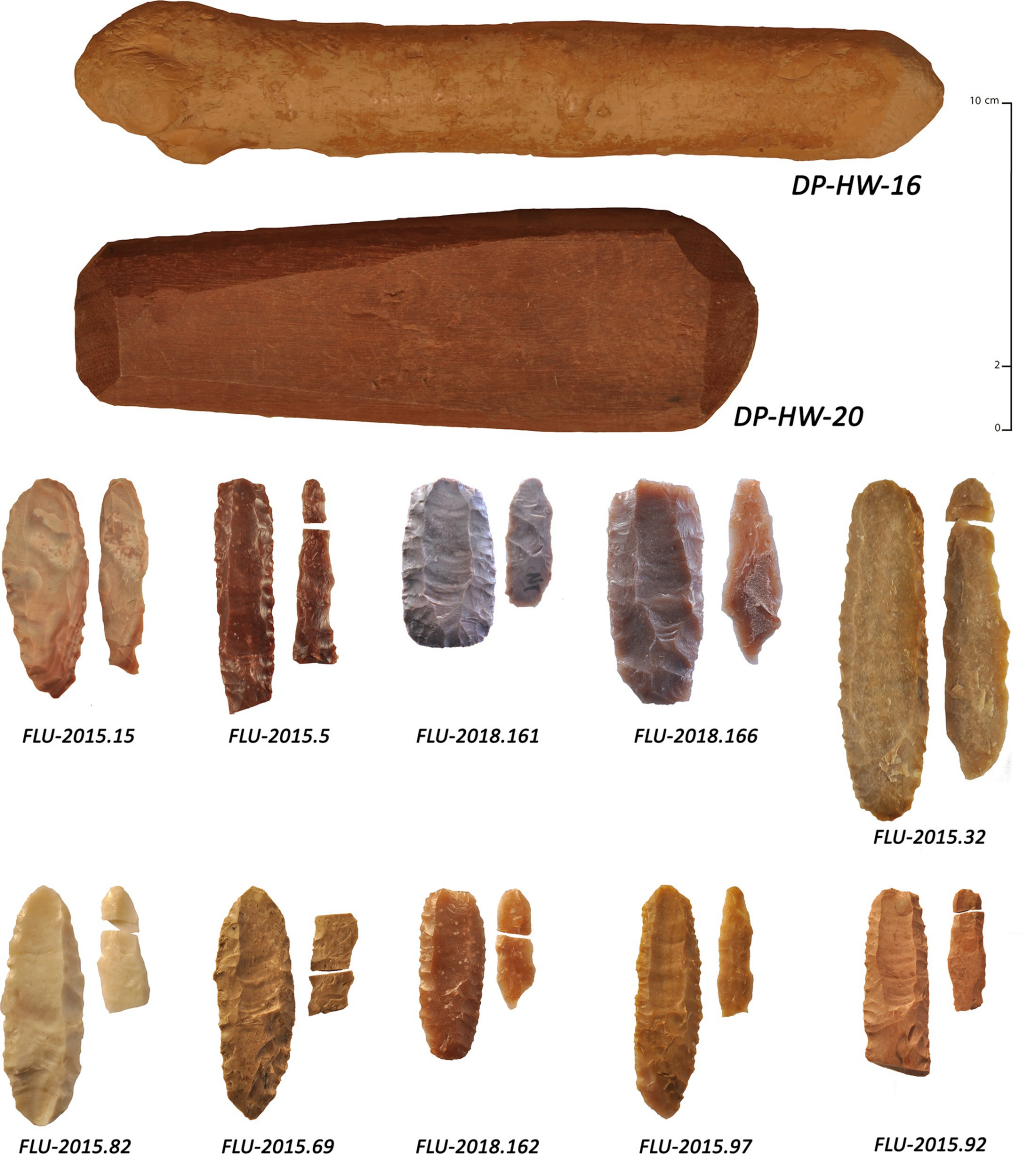
Key results of experimental fluting by direct percussion with hard wood. Top and right: fluted pieces are shown with their channel flake; bottom left: some of the hard woods used in the experiments.

Hard wood hammers weighed from 200 g to 450 g (mean weight of 312 g) and were mainly made of boxwood (*Buxus sempervirens*; density 0.9 to 1.3 kg/dm^3^). We observed relatively thin channel-flakes, with regular thickness and sub-parallel edges and tight ripples on the negative face. The extent of fluting was generally wide (40 mm on average). Complete channel flakes were not rare, but breakages were frequent, often consisting of three fragments, with the medial part being larger. Platforms were faceted, narrow, rarely overflowing and with discrete lips. The bulb was in high position and slightly pronounced. With repeated practice, the knapper managed to significantly limit the hinge and plunging fractures that were abnormally high at the beginning of the experiments. The final experimental tests closely resembled the archaeological finds, including fluting extension, channel flake morphology (length, width and thickness), platform morphology, and fracture types (Fig 15).

**Fig 16:**
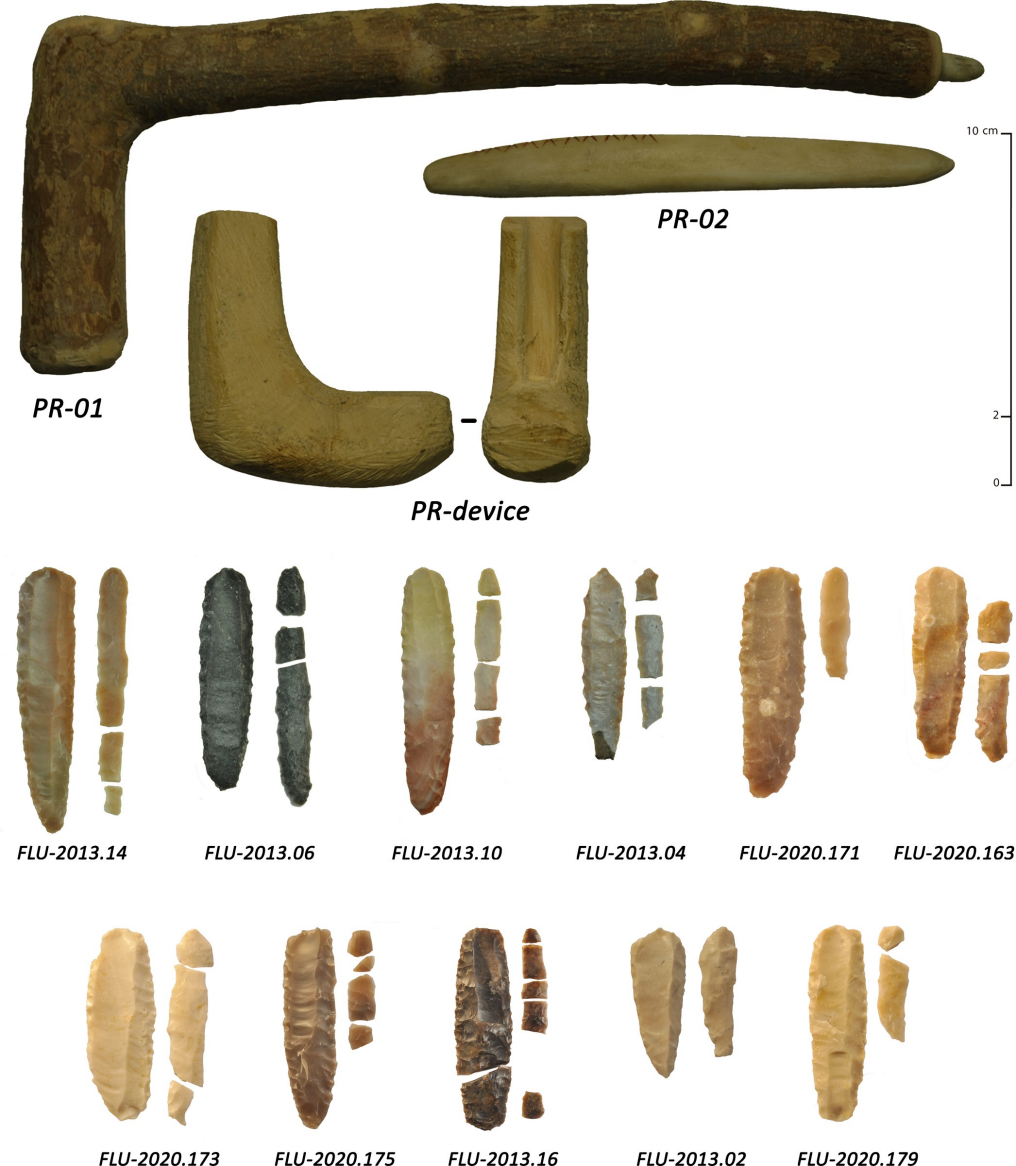
Key results of experimental fluting by pressure. Top: fluted pieces are shown with their channel flake; bottom: some of the tools used in the experiments.

For the pressure detachment technique as applied to fluting, we used red deer antler sticks and a short crutch, including the use of red deer antler, bone tips and wooden pieces for the holding of the preform to conduct fluting (Pelegrin’s Modes 1b and 2 [122]). This detachment technique allowed the removal of very thin and regular channel flakes. Their thickness was regular, edges were mostly parallel and slight ripples appeared on the reverse face. It is important to note that the channel flakes were generally quite narrow. Platforms were faceted and narrow, and bulbs were often absent to slightly pronounced, with no evident lip. The extent of fluting was long (39 mm on average) and the breakages were limited in comparison to direct percussion detachment techniques. Although this detachment technique was somewhat more complicated to carry out, it produced good results (Fig 16).

**Fig 17:**
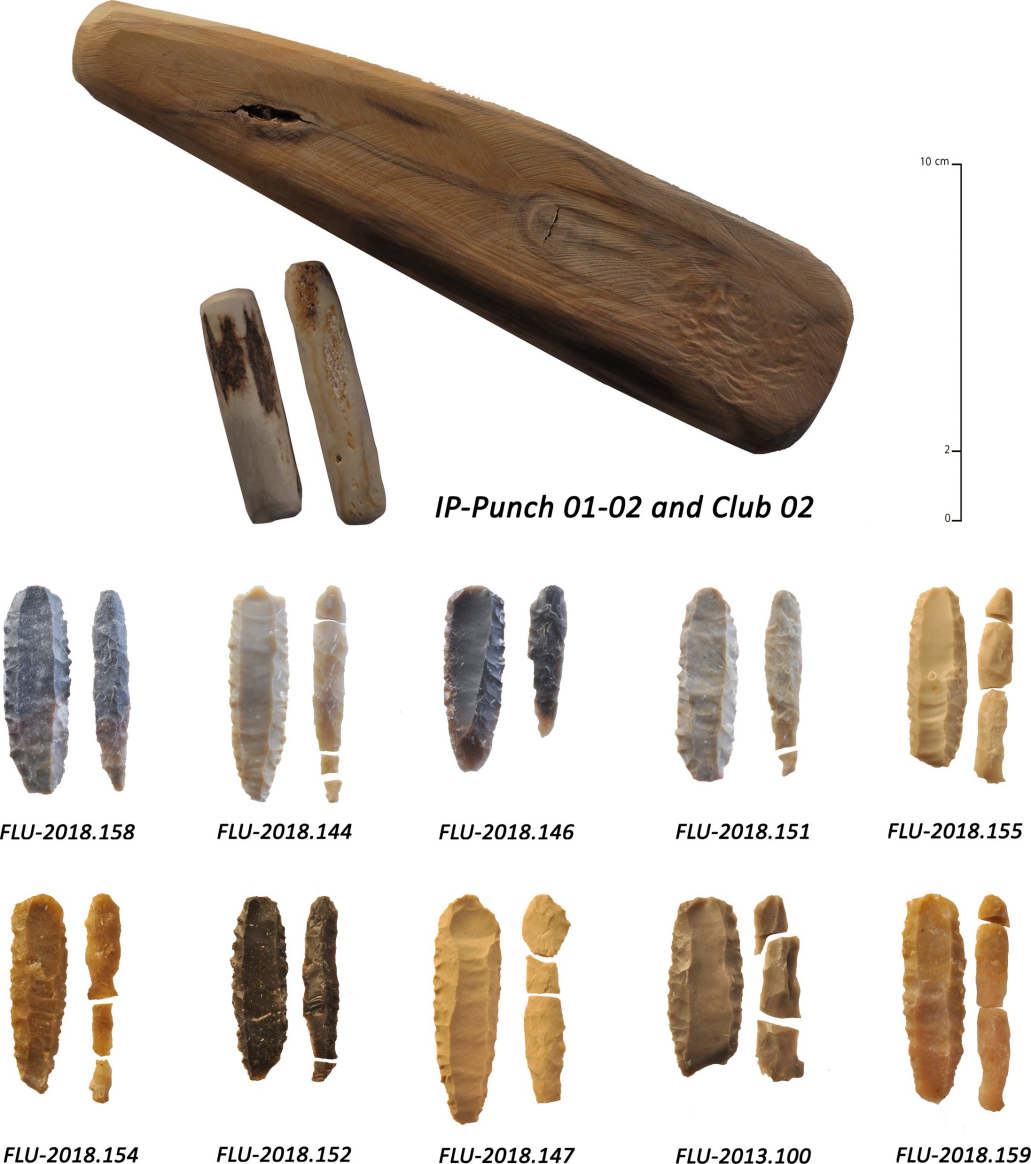
Key results of experimental fluting by indirect percussion. Top: fluted pieces are shown with their channel flake; bottom: some of the tools used in the experiments.

For fluting with the indirect percussion detachment technique, short punches were used, made out red deer antler tines, one slightly curved and one straight and hard wood clubs weighing 245 g and 300 g respectively. Given the small size of the preforms, this detachment technique required grooved pieces of wood as a blocking device. The first attempts were failures but later attempts, with more experience, allowed us to conduct a new series of experiments with more resultant successes. A large number of tests led us to observe that the platforms of channel flakes were frequently large, with slightly pronounced bulbs and lips. Edges were mostly parallel and rectilinear with discrete distal plunging frequently observed. Indirect percussion was part of all the detachment techniques used, allowing the removal of the longest channel flakes (44.5 mm on average). We noted that with a good mastery, it was often possible to flute the entire prepared surface, which is relatively rare among the archaeological series (Fig 17).

### Determining detachment techniques for channel flakes: Comparison between archaeological and experimental data

Determining the detachment technique used for fluting in southern Arabia was considered relevant for better understanding the technological and cultural reasons behind the production of a highly standardized tradition. Channel flakes, from both archaeological contexts and experimental tests, could be used for this purpose.

At Ad-Dahariz 2, the 11 channel flakes were composed of proximal, proximal-mesial and mesial parts (Fig 7). No distal fragments were observed (S1 Table) and no cortex was observed on the 11 pieces. Indications on the nature of the fluting detachment technique can be observed from the channel flakes’ proximal fragments: the presence of a thin lip on the flake’s platform and the remains of the edge of a bifacial striking platform, which is typical of a bifacial thinning flake, but here on a flat and thin channel flake (e.g. S1 Table, piece no. 36). This example is not typical for pressure flaking but for soft organic/mineral percussion. The channel flakes’ platforms may sometimes show signs of heavy abrading and slight crushing (e.g. S1 Table, piece no. 37), probably due to percussion impact, intervening on the edge of the bifacial piece. The mode of preparation implies intense abrading of the platform, which can be associated with fluting made by pressure or soft percussion (with an organic of soft-stone hammer).

The high number (21 in total, including 19 in stratigraphic context and two on the surface) of channel flake fragments (proximal, proximal-medial, medial, medial-distal, and distal; S2 Table) found at Manayzah indicates an *in situ* production of fluted points. Observation of the platforms on proximal parts of the channel flakes indicates frequent “*en éperon*” (spur) preparation, and the bulb is particularly well-outlined and marked. This tends to favor an interpretation for the application of pressure flaking. The systematic softening (strong abrading) of the platforms, sometimes to the point of polishing, strengthens this interpretation. Such preparations avoided shattering the striking platform during extraction using this detachment technique.

It appears that some knappers thermally treated some bifacial tools before fluting. Thermal treatment would be an aid in extracting elongated channel flakes. The presence of a shiny surface detected on channel flakes suggests as much, but we cannot confirm heat-treatment without microscopic analyses. Researchers have in fact observed convincing evidence for heat treatment on chert in Arabia in order to ameliorate the clastic properties of the materials [5,113,123].

**Fig 18:**
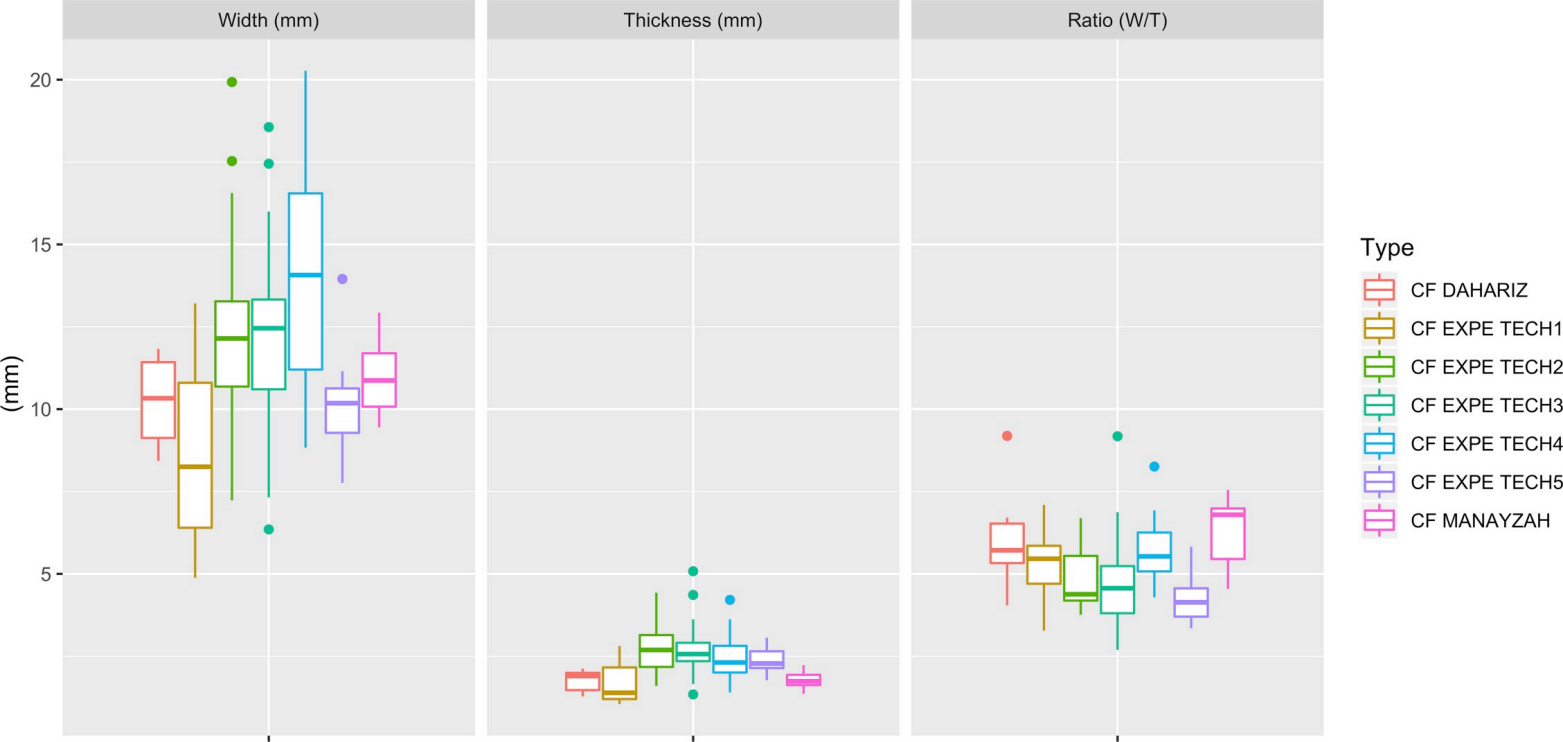
Comparisons of maximum width, maximum thickness and (max. width / max. thickness) ratios between channel flakes from the Manayzah and Ad-Dahariz 2 sites and from the experimental data. Detachment techniques are, 1: pressure, 2: direct percussion with soft stone, 3: direct percussion with antler, 4: direct percussion with wood, 5: indirect percussion.

To carry out meaningful comparisons with archaeological examples, a total of 89 channel flakes was selected among the experimental series of 226 fluting tests (S3 Table). A representative sample of each of the technical categories was achieved: 13 for pressure (14.5%), 20 for direct percussion using soft stone (22.5%), 24 for direct percussion using antler (27%), 15 for direct percussion using hard wood (17%) and 17 for indirect percussion (19%). To evaluate the similarities and differences between the experimental and archaeological channel flakes, a one-way analysis of variance (ANOVA) was performed, testing the null hypothesis that the means and ratios were equal. The data from two archaeological sites and five experimental detachment techniques were compared (“Expe Tech” 1: pressure, 2: direct percussion with soft stone, 3: direct percussion with antler, 4: direct percussion with wood, 5: indirect percussion) (Fig 18). The Levene test of homoscedasticity [F (6.4) = 0.594, p-value = 0.734], and normality checks were carried out and the assumptions met. The ANOVA results indicated statistically significant difference [F(6, 104) = 6.478, p-value = 0.000] among experimental and archaeological channel flakes. This last result led to an a-posteriori analysis using multiple comparison method to determine which means differ from each other. A Tukey’s HSD (Honestly Significant Difference) test was applied for the pairwise comparison for all possible pairs (Table 1).

**Table 1:** Simultaneous tests for general linear hypotheses multiple comparisons of means: Tukey contrasts (codes: 0 ‘***’ 0.001 ‘**’ 0.01 ‘*’ 0.05 ‘.’ 0.1 ‘ ‘ 1). Keys: CF = channel flake; Expe Tech = pressure technique; Expe Tech2 = direct percussion with soft stone; Expe Tech3 = direct percussion with antler; Expe Tech4 = direct percussion with wood; Expe Tech5 = indirect percussion.

Linear Hypotheses	Estimate	Std. Error	t value	Pr(>|t|)
CF Manayzah—CF Dahariz = 0	0.2501	0.4702	0.532	0.99827
CF Expe Tech1—CF Dahariz = 0	-0.6778	0.4517	-1.501	0.74090
CF Expe Tech2—CF Dahariz = 0	-1.1713	0.4139	-2.830	0.07821
CF Expe Tech3—CF Dahariz = 0	-1.3087	0.4015	-3.260	0.02386 *
CF Expe Tech4—CF Dahariz = 0	-0.2319	0.4377	-0.530	0.99831
CF Expe Tech5—CF Dahariz = 0	-1.7169	0.4267	-4.024	0.00207 **
CF Expe Tech1—CF Manayzah = 0	-0.9280	0.4517	-2.054	0.38385
CF Expe Tech2—CF Manayzah = 0	-1.4214	0.4139	-3.434	0.01424 *
CF Expe Tech3—CF Manayzah = 0	-1.5588	0.4015	-3.883	0.00325 **
CF Expe Tech4—CF Manayzah = 0	-0.4820	0.4377	-1.101	0.92524
CF Expe Tech5—CF Manayzah = 0	-1.9670	0.4267	-4.610	< 0.001 ***
CF Expe Tech2—CF Expe Tech1 = 0	-0.4934	0.3928	-1.256	0.86817
CF Expe Tech3—CF Expe Tech1 = 0	-0.6309	0.3797	-1.661	0.63938
CF Expe Tech4—CF Expe Tech1 = 0	0.4459	0.4178	1.067	0.93515
CF Expe Tech5—CF Expe Tech1 = 0	-1.0391	0.4063	-2.558	0.14792
CF Expe Tech3—CF Expe Tech2 = 0	-0.1374	0.3339	-0.412	0.99960
CF Expe Tech4—CF Expe Tech2 = 0	0.9394	0.3766	2.494	0.16941
CF Expe Tech5—CF Expe Tech2 = 0	-0.5456	0.3638	-1.500	0.74120
CF Expe Tech4—CF Expe Tech3 = 0	1.0768	0.3629	2.967	0.05493
CF Expe Tech5—CF Expe Tech3 = 0	-0.4082	0.3496	-1.168	0.90306
CF Expe Tech5—CF Expe Tech4 = 0	-1.4850	0.3906	-3.802	0.00433 **

Table 1 shows that the significant difference between means ratio is identified between:

• CF Expe Tech5 and CF Manayzah (t = -4.610, p-value = 0.001 ***)

• CF Expe Tech5 and CF Dahariz (t = -4.024, p-value = 0.00207 **)

• CF Expe Tech3 and CF Manayzah (t = -3.883, p-value = 0.00325 **)

• CF Expe Tech3 and CF Dahariz (t = -3.260, p-value = 0.02386 *)

• CF Expe Tech2 and CF Manayzah (t = -3.434, p-value = 0.01424 *)

• CF Expe Tech4 and CF Expe Tech5 (t = -3.802, p-value = 0.00433 **)

**Fig 19:**
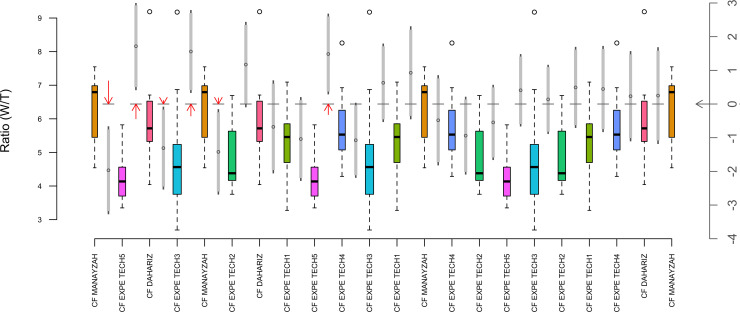
Boxplots with pairwise testing of (width / thickness) ratios channel flakes from the Manayzah and Ad-Dahariz 2 sites and the experimental data. Detachment techniques are 1: pressure, 2: direct percussion with soft stone, 3: direct percussion with antler, 4: direct percussion with wood, 5: indirect percussion. Variable width of the boxplots reflects group sizes. The gray vertical strip between each pair of boxplots depicts the HSD confidence interval for the difference in means from the distributions in the two boxplots. The circle in the middle of the gray strip is the point estimate for the difference in means. The axis on the left-hand side refers to the boxplots, the right-hand axis refers to the confidence intervals. Two levels of confidence are depicted by the gray strips. The light grey part shows a 90% interval. Moving out to include the mid-gray section gives a 95% interval. The horizontal gray line segment shows the zero value of the right hand or confidence interval axis. The red arrows depict comparisons where the confidence interval does not intersect the zero axis. Longer arrows mean greater significance, i.e. smaller p-values.

No statistical difference was detected between the remainder of the comparison pairs. Fig 19 illustrates another way to interpret these results by visualizing the confidence intervals and the boxplots with pairwise testing (width / thickness) of ratios. Considered together, the results indicate that there is a significant difference between some techniques and sites, and this significant difference is specifically located between six pairs of comparison. These results demonstrate that channel flakes from Ad-Dahariz and Manayzah are not statistically different (t = 0.532, p-value = 0.99827). It should be noted that the techniques used to produce the channel flakes CF Expe Tech5 (indirect percussion) and CF Expe Tech4 (direct percussion with wood) are unique techniques that are statistically different (t = -3.802, p-value = 0.00433 **).

Finally, and importantly, there is a significant difference between channel flakes of Expe Tech3 (direct percussion with antler), Expe Tech5 (indirect percussion) and the two archaeological sites Manayzah and Ad-Dahariz (Table 1). Therefore, the two experimental techniques differ from the techniques used on the two sites. However, Expe Tech1 (pressure technique) and Expe Tech4 (direct percussion with wood), and to a lesser extent Expe Tech2 (direct percussion with soft stone), show no significant differences with the archaeological data (Table 1). This indicates a similarity in the fluting detachment techniques between the experimental and archaeological data.

The observations gained by experimentation is supported by the statistical tests, leading to the conclusion that pressure and/or direct percussion (with hard wood and/or soft stone) techniques were most probably used by the Neolithic knappers in southern Arabia for fluting.

## Discussion

### Function, level of expertise and cultural transmission

Experiments have highlighted the value of the direct percussion technique, although this detachment technique is difficult to master. It is also slightly less efficient than pressure or indirect percussion in creating large and regular surfaces. Even if it seldom removes the whole fluted surface, direct percussion fluting still removes a large and regular surface between one-third to two-thirds of the anticipated arrowhead, with a very discreet negative bulb and light undulations. These parameters greatly facilitate a later reworking by pressure for a very regular and correct trihedral shaping. It can thus be seen that a thorough exploration of traditional ecological knowledge (or more simply expertise), linked to an observed technical register, led to the emergence of a complex *chaine opératoire* whose purpose is linked to consistent criteria and constraints of lightness and/or robustness of the projectile tips.

As noted above, North American fluting is closely linked to the hafting of projectiles. This technique provides a skillful response to the robustness/lightness constraint of the projectile tip, because the tip is made lighter by the fluting while maintaining robust edges. In South Arabia, the robustness/lightness constraint of the projectile tip is resolved by trihedral shaping. Here, fluting provides an added value that reinforces the regularity of the tip in section (shaping fluting). The American and Arabian cases thus diverge in terms of final forms, but both testify to a remarkable investment in projectile tip technology.

The lithic experiments provided a context to further consider the functional interpretation of fluting. Observations of the archaeological series of Arabian trihedral fluted points, fluted blanks and channel flakes confirm that fluting removals cannot be the result of accidental impacts. During replication of the Ad-Dahariz fluted point types (more tapered and elongated than the Manayzah type), the fluted surface was used as a flat and regular pressure platform. This flat morphology greatly facilitated the trihedral shaping of the point. One can then consider for the Ad-Dahariz type that fluting is not an end, but a component of a trihedral shaping method. This interpretation, however, does not apply to the Manayzah fluted points or other tanged fluted points observed elsewhere in South Arabia, as the fluted area is not used as a striking platform, and was untouched in the reduction process. Nevertheless, the presence of unfluted trihedral points at Ad-Dahariz appears similar to the fluted ones and this is a rather surprising aspect of the assemblage, pointing to a non-functional interpretation of fluting in Arabia. In fact, both Arabian fluting types, i.e. the tanged and wider points from Manayzah and the tempered points from Dhofar, do not provide convincing reasons of fluting for hafting reasons or because a fluted surface would have enhanced the shaping process. An interpretation towards a demonstration of skill, a costly signaling tradition, or a sociocultural role is therefore reasonable to consider.

Experiments have made it possible to highlight the technical expertise involved in each stage of the manufacturing process and to assess the level of difficulty in manufacturing processes. We were able to confirm the high level of expertise that these production techniques required. Despite regular practice and correct level of expertise in flintknapping, it took us several years of intermittent practice and more than two hundred attempts to assimilate and master the gestures of fluting and trihedral shaping. This expertise is acquired, and in the case of Neolithic societies through apprenticeship, and reinforced by regular and sustained practice. The community therefore invested time and energy in forming expert-craftsmen. This implies a special place for technical expertise within the group and it is recognized as a potential sign of the expertise of an individual within a group, and of the quality of a group within a wider community.

The transmission of specific expertise is a transmission of the cultural values of the community. Technical expertise is an essential value because it is transmitted at the cost of a significant investment in learning and practice (requiring raw materials, tools and time). The transmission of such skill related to fluting and trihedral shaping within the group allows the perpetual production of a method that is a compromise between lightness and robustness related to the “ballistic” efficiency of arrowheads. Whether dedicated to hunting activities or resolving inter-community conflicts, the use of these techniques confers a definite advantage. There may therefore be little interest for the group to disseminate and transmit this knowledge outside the community. However, it is entirely possible that these projectile forms can be copied or imitated. This may help to explain why some of them are not fluted, or why some groups have developed parallels covering retouching linked to trihedral shaping rather than fluting. The idea of the trihedral arrowhead could circulate independently of the modalities of its realization.

### Convergent evolution of the fluting method in the Americas and Arabia

Here we emphasized the technical and methodological processes involved in fluting rather than on the resultant end forms. Consequently, what we call fluting here is the method of knapping, and not the final aim of the action, such as emphasis on hafting in American examples. The discovery of the same invention in two widely separated regions and at two different time periods raises a number of questions. As most archaeologists dealing with fluted points will likely quickly observe, fluted points in America are typologically and functionally different compared to those in Arabia. Therefore, is it legitimate to use the same terminology to call Arabian fluted points “fluted”? The problem surrounding the definition of fluting has been raised previously, leading some to differentiate between fluted points and a technique for re-thinning or re-sharpening points (e.g. [84,124,125]). Consensus suggests that a strict typological analysis of fluted points is insufficient, however, owing to archaeologists need to understand intent, which is difficult to quantify. Re-stated, is fluting a real and common feature between America and Arabia, or is it a constructed concept by archaeologists? And can we really and clearly define a “fluting method”?

Our experiments, based on the South Arabian fluted points, confirm intention in fluting small bifaces. Insofar as we can reproduce the Arabian fluted points, application of a strict *chaine opératoire* is critical in reproducing a fluted tool. We chose to call them fluted points, even as we highlight some differences in the processes between the Americas and Arabia. Indeed, the main difference is in the sequencing of the *chaines opératoires*, as fluting intervenes at the end of the shaping of the point in America, while it appears at the mid-point of the shaping of the lithic point in Arabia. In the American literature, the fluting method often strictly refers to the preparation of a hafting zone. However, here we define a fluting method that can also be applied from the proximal end or tip of a piece. Moreover, the production of thin bifaces is not necessary in the preliminary steps of the *chaine opératoire*, as the blanks themselves can range from trihedral items to various other forms. In the Arabian case, we expand the technical and cultural understanding of fluting, making it impossible to comprehensively link these expressions to a single cultural tradition or adaptive strategy. Instead, fluting offers insights into the issue of convergence (or convergent evolution) in form across different regions without any sign of cultural transmission. This encompasses more than what a strict American definition would allow.

The present study links the Americas and Arabia by addressing to what degree cultural transmission accounts for convergence in knapping technology. We demonstrated that the fluting method appeared in Arabia as an independent invention. The only possible comparison with Arabia, at a worldwide scale, comes with older and far-distant American examples.

For decades, lithic industries throughout the world have been studied in a multitude of ways, including examination of human dispersals at an intercontinental scale, analysis of economies at a regional scale, and activity variation at the site scale. Lithic industries have been viewed as evidence of human technological and cognitive abilities and human groups’ cultural heritage through time and space. Studying lithic industries through a convergent evolution approach is a more recent topic of interest [126,127], and an extremely important one, as studying culture in an evolutionary framework allows the development of models about the pattern of human cultural diversity and various forms of human adaptation.

The concept of convergent evolution was originally developed in the biological sciences [128], explaining similar or comparable processes that occurred in independent lineages, and resulting from various constraints. By identifying convergence in fluting in the Americas and Arabia, with no demonstrable cultural contact, we demonstrate a clear illustration of independent invention that did not involve any physical or cultural connection among toolmakers.

By definition, lithic production encompasses an inevitable reduction of volume, a core in the case of blade or flake production, a blank in the case of a bifacial thinning, and a small biface in the case of fluting. As the volume of stone is reduced, the flintknappers face a diminution of possibilities in reduction and choices in the shaping of the predetermined desired object. Individual choices are inexorably driven by physical constraints in the fracturing properties of stone. All these aspects can increase the possibilities for convergence of lithic technologies. However, fluting itself occurs in the Americas and in Arabia for perhaps different reasons. There is convergence in the flintknapping method itself, but not necessarily in the functional or non-utilitarian objectives. Paleoindian populations and Arabian Neolithic groups may have faced comparable adaptive challenges, influencing the independent invention of fluting. However, the fluting method in the Arabian Neolithic may also involve a heightened degree of cultural evolution, or “hypertely” (an exaggerated specialization, an extreme functional over-adaptation), *sensu* [129–131]. That said, our definition fits well with a universally applicable concept, which is increasingly observed in various lithic assemblages [126,127,132,133], and is consistent with evolutionary approaches in understanding the case of fluting as a socio-cultural process [21,134] at different times and in different places.

### Interpretation of fluting in South Arabia

Given interpretations for hafted fluted forms in America, and their technical contrasts with Arabian fluted forms, we can address whether the differences in technology equate to differences in intent. As previously indicated, multiple interpretations surround the fluted forms in the Americas, with most researchers centering on a functional explanation in hafting performance. In comparing American fluted points to Arabian ones, the position and nature of the flutes differ. American points tend to be systematically fluted from the proximal end of the armature, and often on both sides, but in South Arabian forms, fluting is also conducted from the proximal end, indicating that other, non-functional explanations must be explored.

On the one hand, fluted points from Manayzah and more broadly from southern Arabia were obviously used as projectile points, most probably arrowheads. The fluting method itself renders the weapon lighter, offering a substantial kinetic improvement. We demonstrated that knappers at Manayzah tried to obtain fluting scars of a considerable length. They abandoned unfinished tools that were not sufficiently fluted. The Ad-Dahariz type is more robust, the flute maybe permitted to facilitate the thinning of the triangular section changing a rather fragile piece into a small and solid projectile point. On the other hand, the fluting process in Arabia mostly occurred from an arrowhead’s tip. In such cases, the fluted zone cannot be interpreted as a surface to facilitate hafting and no real functional explanation can be found. It is possible therefore reasonable to suggest that fluting gestures are part of a display of skills (*contra* [3]) or a display of technical dexterity by highly specialized expert-craftsmen or ‘flintknapping masters’. Indeed, fluting preparation and success involve an accumulation of knowledge and significant commitment and investment to acquire the skill. Moreover, fluting represents a risky operation, regularly ending with breakage of time-costly bifacial preforms.

Did the fluting method in Arabia and its successful implementation convey important cultural information? We tentatively suggest that technical knapping skill signaled a high level of cultural expertise linked to hunting and served as costly signaling of unobservable qualities that ensured individual success. A potentially wasteful display of expertise, fluting may nonetheless signal another expertise: prowess in hunting game while defending one’s own territory or herd. There was care lavished on delicate tangs and futile fluting, both details that show individual skill but convey little adaptive advantage in the physical requirements of hunting or defense.

Manayzah’s herding people have been interpreted as hunters and herders [115]. Although the faunal collections at Manayzah are small, the assemblage includes mostly gazelle and caprines, the latter of which may or may not belong to domesticates. There are only ten cattle represented alongside one sheep bone. In short, if these were herders who tended rather than poached domesticates, they relied on wild prey, perhaps for most of their meat [114]. And this suggests that hunting, along with the costly signaling (ethnographically suggested to explain the sharing of large meat packages) [135], plays an important role in understanding the knapping behavior of South Arabian Neolithic herders. We find evidence of highly skilled knappers at Manayzah, Ad-Dahariz and at other sites with fluted points in South Arabia. Individual prowess may be a means by which people mediated social relationships in this early Neolithic phase of Southern Arabia [116].

In Arabian prehistory, South Arabia experienced a rather autochthonous development during the Neolithic, with multiple examples of inventions and innovations not culturally transmitted by other traditions from outside [6,7,107,117,118]. The fluting method is then a hallmark of this indigenous development in the South Arabian Neolithic.

## Conclusion

This overview of both American and Arabian fluting technologies allows us to demonstrate the intention and success of fluting in Arabia as a purely independent invention millennia after it was originally invented across the Atlantic Ocean. We propose a new perspective in American assemblages’ approach, as what we call fluting in Arabia does not necessarily equate with the same technology in the Americas. We emphasized that the ‘fluting method’ is a mental conceptualization of knapping, more than just a technical way to produce a hafting zone, as most of the Arabian fluted points are fluted but do not hafting as a functional final aim. The fluting concept and the method itself are the same in both regions, yet the ways to achieve it may be different.

The interpretation of the function and objectives of fluted point technology in Arabia is difficult to ascertain, though this remains a challenging and thought-provoking research endeavor. The origin, expansion and disappearance of the fluting method in Arabia still needs to be documented in detail with further archaeological field work. Given what we know at present, fluted forms are geographically constrained to the south-central and south-east parts of the Arabian Peninsula and it never spread out from there, providing a significant contrast with the Americas where fluting was geographically widespread and occurred over a longer period of time. The fluting method was a highly refined and sophisticated technology that was developed and used in a limited area of southern Arabia, and it is without parallel elsewhere in the Neolithic world. In addition to explanations identifying fluting as a functional development to enhance tool efficiency, or as a marker of cultural identity, we suggest that knapping prowess in socio-cultural context may offer a reasonable explanation.

## Supporting information

S1 Table Dimensions of channel flakes from Ad-Dahariz 2. (PDF)

S2 Table Dimensions of channel flakes from Manayzah. (PDF)

S3 Table Experimental corpus of fluted points used in this study. (PDF)
