# Supplementary material for: Fluted-point technology in Neolithic Arabia: An independent invention far from the Americas
Source: PLoS One. 2020 Aug 5;15(8):e0236314. doi: 10.1371/journal.pone.0236314 (PMC7406013; doi:10.1371/journal.pone.0236314)
Supplement: S1 Table — (PDF) [file pone.0236314.s001.pdf]

S1 Table. Dimensions of channel flakes from Ad-Dahariz 2.

AREA	Piece #	max. length (mm)	max. width (mm)	max. thick. (mm)	weight (g)	State	Butt
general surface	**5**	11,45	8,43	1,32	0,1	proximal	punctiform
general surface	**6**	11,25	9,74	1,98	0,2	medial	
2H (surface)	**7**	12,77	11,76	1,28	0,2	medial	
2H (surface)	**8**	14,79	10,33	1,54	0,2	medial	
2H (surface)	**9**	16,73	10,39	1,9	0,4	proximal-medial	abraded/crushed
2H (surface)	**10**	19,83	11,49	2,01	0,5	proximal-medial	punctiform
Test pit -10cm	**11**	18,89	9,33	1,4	0,2	proximal-medial	punctiform
general surface	**36**	13,73	11,83	1,91	0,2	proximal-medial	facetted
general surface	**37**	11,89	8,92	1,71	0,2	proximal-medial	abraded/crushed
general surface	**38**	12,8	11,36	2,09	0,4	medial	
general surface	**127**	8,82	8,57	2,12	0,1	medial	
