# Supplementary material for: Fluted-point technology in Neolithic Arabia: An independent invention far from the Americas
Source: PLoS One. 2020 Aug 5;15(8):e0236314. doi: 10.1371/journal.pone.0236314 (PMC7406013; doi:10.1371/journal.pone.0236314)
Supplement: S2 Table — (PDF) [file pone.0236314.s002.pdf]

S2 Table. Dimensions of channel flakes from Manayzah.

AREA	Piece #	max. length (mm)	max. width (mm)	max. thick. (mm)	State	Butt
A-M8-001	**1**	19,13	12,93	1,74	proximal	punctiform
A-L9-001	**1**	31,87	12,91	1,89	medial-distal	
A-M8-002	**1**	20,37	10,02	2,08	proximal	punctiform
A-M8-002	**2**	19,53	11,54	1,97	medial	
A-ALL-005	**1**	18,66	10,13	2,23	proximal	punctiform
A-M8-010	**1**	14,33	9,56	1,36	proximal	punctiform
A-L9-011	**1**	15,07	10,33	1,9	proximal	punctiform
A-L9-011	**2**	14,09	9,45	1,73	proximal	punctiform
B-ALL-001	**1**	27,91	11,45	1,65	proximal-medial	punctiform
B-ALL-001	**2**	23,82	10,87	1,6	medial-distal	
D-L12-001	**1**	18,93	11,85	1,57	distal	
