# Supplementary material for: Fluted-point technology in Neolithic Arabia: An independent invention far from the Americas
Source: PLoS One. 2020 Aug 5;15(8):e0236314. doi: 10.1371/journal.pone.0236314 (PMC7406013; doi:10.1371/journal.pone.0236314)
Supplement: S3 Table — (PDF) [file pone.0236314.s003.pdf]

S3 Table. Experimental corpus of fluted points used in this study

N°	date	R.M.	H.T.	Shap.	Fl. T	Tool	Hold.	Fl. org.	C-F nbr.	Fgts nbr.	Lack	C-F Acc.	C-F dist.prof.	Butt	Bulb	Lip	Bb. sc.	Rip. Am. C-F	Rip. Loc. C-F	Edg. Par. C-F	Edg. Reg. C-F	C-F ov.fl.	F.P. Acc.	l. F.P mm	w. F.P mm	th. F.P mm	Fl. l. mm	Fl. Lght %	l. C-F mm	w. C-F mm	th. C-F mm	observations / remarks
**1 pressure**																																
**FLU-1**	2013	1	0	1	1	1	3	1	1	4	4	4	3	2	2	1	1	1	3	1	2	0	0	40	18,6	6,3	40	100	43,30	10,90	2,16	
**FLU-2**	2013	1	0	1	1	1	3	1	1	2	3	4	3	2	2	1	1	1	3	2	1	0	0	41,2	15,5	6	41,2	100	36,20	10,80	2,60	
**FLU-3**	2013	2	1	1	1	2	3	1	1	4	0	0	1	2	2	1	1	1	3	2	1	0	0	50,6	14,7	10,7	32,4	64	28,64	5,64	1,20	
**FLU-4**	2013	2	1	1	1	2	3	1	1	3	0	0	1	2	2	1	1	1	3	2	1	0	0	55	15,2	8,1	43,4	79	43,00	9,08	1,28	
**FLU-5**	2013	1	1	1	1	3	4	1	1	5	0	6	1	2	1	2	1	1	3	2	3	0	0	74,6	17,5	9,2	74,6	100	73,34	13,21	2,42	
**FLU-6**	2013	9	1	1	1	3	4	1	1	3	0	4	3	4	2	1	1	2	3	1	1	0	0	65,4	17,9	8	65,4	100	69,17	11,52	2,81	
**FLU-7**	2013	3	1	1	1	3	4	1	1	3	0	5	4	0	0	0	0	2	3	3	1	0	1	61,13	16,8	8,8	0	0				
**FLU-8**	2013	3	1	1	1	3	4	2	1	3	2	2	2	2	1	2	1	2	3	2	2	0	3	59,4	19,5	7,2	24,4	41				double fluting
**FLU-8-CF2**	2013	3	1	1	1	3	4	2	1	3	4	2	2	2	2	2	1	1	3	3	1	0		59,4	19,5	7,2	35,5	60				
**FLU-9**	2013	3	1	1	1	3	4	1	1	3	0	5	4	1	2	2	1	0	0	3	1	0	1	66	16,35	6,7	0	0				
**FLU-10**	2013	3	1	1	1	3	4	1	1	4	0	2	2	1	2	1	1	1	3	1	1	0	0	73,1	19,4	6,7	46,6	64				
**FLU-11**	2013	3	1	1	1	3	4	2	1	2	1	2	2	0	0	0	0	3	3	1	1	0	2	64,6	18,6	8	24,8	38				double fluting
**FLU-11-CF2**	2013	3	1	1	1	3	4	2	1	2	1	2	2	0	0	0	0	1	3	1	1	0		64,6	18,6	8	34,8	54				
**FLU-12**	2013	4	1	1	1	3	4	1	1	4	0	4	3	0	0	0	0	1	3	1	1	0	0	35,5	17,6	5,7	35,5	100	35,98	8,25	1,49	
**FLU-13**	2013	5	1	1	1	3	4	1	3	8	0	5	4	1	2	1	1	2	3	3	1	0	1	58,6	15,6	6,2	0	0				
**FLU-14**	2013	6	1	1	1	3	3	1	1	3	3	3	2	3	2	1	2	1	4	1	1	0	0	76,1	17,9	7,7	66,66	88	59,18	9,20	1,39	
**FLU-15**	2013	7	1	1	1	3	3	3	1	1	0	0	1	1	2	1	1	1	4	2	1	0	0	48,8	16,9	7,4	17,6	36	29,00	6,40	1,36	quadruple fluting
**FLU-15-CF2**	2013	7	1	1	1	3	3	3	1	2	0	0	1	1	2	1	1	1	3	2	1	0		48,8	16,9	7,4	18,2	37	22,58	6,09	1,05	
**FLU-15-CF3**	2013	7	1	1	1	3	3	3	1	1	3	0	1	1	2	1	1	1	3	2	1	0		48,8	16,9	7,4	21	43	18,37	4,88	1,49	
**FLU-15-CF4**	2013	7	1	1	1	3	3	3	1	2	0	0	1	1	2	1	1	1	3	2	1	0		48,8	16,9	7,4	29,4	60	20,86	6,73	1,15	
**FLU-16**	2013	4	1	1	1	3	4	1	1	6	0	2	2	1	2	2	1	0	0	1	1	0	7	42,5	16,8	7	28	66	28,30	7,18	1,06	
**2 direct percussion using soft stone**																																
**FLU-17**	2013	8	1	1	2	5	1	1	1	0	0	3	5	1	2	1	1	2	3	4	2	0	0	82,4	20,2	15,6	51	62	50,71	19,93	3,40	
**FLU-18**	2013	7	0	1	2	5	1	1	1	0	0	0	1	1	2	1	2	1	3	3	2	0	0	66,3	17,6	11,8	17,8	27				
**FLU-19**	2013	7	0	1	2	5	1	2	1	1	0	1	2	2	2	1	1	1	4	3	2	1	3	47	15,7	10,2	11	23				double fluting
**FLU-19-CF2**	2013	7	0	1	2	5	1	2	1	1	0	2	2	1	2	1	1	0	0	1	1	2		47	15,7	10,2	21,3	45				
**FLU-20**	2013	7	0	1	2	5	1	1	1	2	3	2	2	1	2	1	1	2	3	1	2	0	0	61,5	17	7,4	34,5	56				
**FLU-21**	2013	1	0	1	2	5	1	1	1	0	0	0	1	2	3	2	1	1	3	1	2	0	0	54,1	19,1	6,9	33	61				
**FLU-22**	2013	7	0	1	2	5	1	2	1	2	0	2	2	4	1	1	2	1	3	1	2	0	3	70,5	18,2	9,8	33	47				double fluting
**FLU-22-CF2**	2013	7	0	1	2	5	1	2	1	2	0	2	2	1	2	1	1	1	3	4	3	0	2	70,5	18,2	9,8	28,8	41				
**FLU-23**	2013	1	0	1	2	6	1	2	1	1	0	1	2	3	2	1	1	0	0	3	2	2	1	54	14	9	0	0				double fluting
**FLU-23-CF2**		1	0	1	2	6	1	2	1	1	0	5	4	2	1	2	1	0	0	1	1	0		54	14	9	14,8	27				
**FLU-24**	2013	1	0	1	2	6	1	2	1	1	0	2	2	2	2	1	1	1	3	2	2	0	3	46,3	16,1	8,3	26,9	58				double fluting
**FLU-24-CF2**	2013	1	0	1	2	6	1	2	1	1	0	2	2	1	2	1	2	0	0	3	1	0		46,3	16,1	8,3	17,3	37				
**FLU-25**	2013	1	0	1	2	6	1	1	1	0	0	1	2	2	2	1	1	0	0	3	1	0	2	53,4	14,7	8,2	18,4	34				
**FLU-26**	2013	5	1	1	2	6	1	2	1	2	0	1	2	1	1	1	1	1	3	1	2	0	2	64,6	15,1	8,2	11,4	18				double fluting
**FLU-26-CF2**	2013	5	1	1	2	6	1	2	1	1	0	2	2	3	2	1	1	0	0	3	2	0		64,6	15,1	8,2	24,4	38				
**FLU-27**	2013	2	0	1	2	6	1	2	1	2	1	1	2	0	0	0	0	0	0	1	1	0	2	48,6	14	6,5	12,5	26				double fluting
**FLU-27-CF2**	2013	2	0	1	2	6	1	2	1	0	4	1	2	3	2	1	1	0	0	1	2	0		48,6	14	6,5	8,2	17				
**FLU-28**	2013	1	0	1	2	6	1	2	1	1	0	1	2	4	2	1	1	0	0	3	1	0	3	41,6	15,2	6,9	11,2	27				double fluting
**FLU-28-CF2**	2013	1	0	1	2	6	1	2	1	1	0	0	1	3	3	1	1	1	3	1	2	0		41,6	15,2	6,9	25,5	61				
**FLU-29**	2013	6	1	1	2	6	1	1	1	3	1	5	4	0	0	0	0	3	3	3	2	0	1	47,9	16,3	6,8	0	0				
**FLU-30**	2013	10	1	1	2	6	1	1	1	0	0	2	5	1	2	1	1	0	0	4	2	0	2	66,5	18,4	16	46,3	70				
**FLU-31**	2013	7	1	1	2	6	1	2	1	1	0	2	2	1	2	1	1	1	3	4	3	0	2	34,4	12,4	7	21,4	62				double fluting
**FLU-31-CF2**	2013	7	1	1	2	6	1	2	1	1	0	5	4	1	2	1	1	0	0	3	2	1	1	34,4	12,4	7	0	0				
**FLU-32**	2013	5	1	1	2	6	1	2	1	1	0	2	2	2	2	1	1	1	3	1	2	0	3	52,4	17,3	11,9	23,8	45				double fluting
**FLU-32-CF2**	2013	5	1	1	2	6	1	2	1	1	3	2	2	1	2	2	1	1	3	1	2	0		52,4	17,3	11,9	20,6	39				

**FLU-33**	2013	6	1	1	2	6	1	2	1	1	0	2	2	4	2	1	1	0	0	2	2	0	3	37,8	16,2	8,3	17,5	46				triple fluting
**FLU-33-CF2**	2013	6	1	1	2	6	1	2	1	1	3	1	2	1	2	1	1	0	0	1	2	0		37,8	16,2	8,3	12,7	34				
**FLU-33-CF3**	2013	6	1	1	2	6	1	2	1	1	0	2	2	1	2	1	1	1	3	4	3	0		37,8	16,2	8,3	19	50				
**FLU-34**	2013	1	0	1	2	9	1	1	1	0	0	2	2	4	2	1	1	1	3	1	3	1	2	53,4	11	9,5	0	0				trihedral shaping on fracture
**FLU-35**	2015	5	1	1	2	6	1	2	1	1	0	2	2	2	2	1	1	0	0	3	2	0	0	57,6	22,3	12	28,3	49				double fluting
**FLU-35-CF2**	2015	5	1	1	2	6	1	2	1	1	0	0	1	1	2	1	1	1	3	3	2	0		57,6	22,3	12	32,4	56				
**FLU-36**	2015	5	1	1	2	6	1	2	1	1	0	3	2	2	3	1	1	1	3	1	3	0	3	53	19,4	12,6	31,1	59				double fluting
**FLU-36-CF2**	2015	5	1	1	2	6	1	2	1	2	4	1	2	3	1	1	1	0	0	1	2	0		53	19,4	12,6	20,5	39				
**FLU-37**	2015	6	1	1	2	7	1	1	1	0	0	5	4	2	2	1	1	3	3	3	3	0	1	79	25,15	15,6	0	0				
**FLU-38**	2015	3	1	1	2	8	2	1	1	0	0	2	2	2	2	1	2	0	0	4	3	0	4	61,5	23,9	10,7	34	55				
**FLU-39**	2015	5	1	1	2	8	1	1	1	3	0	3	5	2	2	1	1	3	3	4	3	0	2	65,5	21,5	11,7	48,1	73	49,49	16,38	3,01	
**FLU-40**	2015	5	1	1	2	8	1	1	1	0	0	3	5	2	2	1	1	3	3	1	3	0	2	69,2	23	12,8	55,6	80				
**FLU-41**	2015	5	1	1	2	8	1	1	1	2	0	5	4	1	2	2	1	0	0	3	2	0	1	50,1	19,6	11	0	0				
**FLU-42**	2015	3	1	1	2	8	1	1	1	0	0	2	2	1	2	1	1	2	3	4	2	0	2	79,2	20,3	9,6	49,2	62				
**FLU-43**	2015	5	1	1	2	8	1	1	1	0	0	5	4	4	2	1	1	0	0	3	1	0	1	57,1	16,5	10,8	0	0				
**FLU-44**	2015	3	1	1	2	10	1	1	1	0	0	5	4	1	2	1	1	2	3	3	2	0	1	70,6	23,9	12,6	0	0				
**FLU-45**	18/06/2015	6	1	1	2	11	2	2	1	1	0	2	2	4	2	1	1	1	3	4	2	0	0	58,4	25,2	10,4	37,9	65				quadruple fluting
**FLU-45-CF2**	18/06/2015	6	1	1	2	11	2	2	1	1	0	0	1	0	0	0	0	0	0	1	1	0		58,4	25,2	10,4	24	41				
**FLU-45-CF3**	18/06/2015	6	1	1	2	11	2	2	1	1	0	0	0	4	2	1	1	0	0	1	3	0		58,4	25,2	10,4	27,3	47				
**FLU-45-CF4**	18/06/2015	6	1	1	2	11	2	2	1	1	0	0	1	1	2	1	1	0	0	3	2	0		58,4	25,2	10,4	32,8	56				
**FLU-101**	2018	3	1	1	2	10	1	1	1	1	0	0	5	2	2	2	1	1	3	1	1	0	0	77,02	22,75	18,18	47,05	61	47,05	17,53	4,43	
**FLU-102**	2018	3	1	1	2	10	1	1	1	1	0	0	1	1	2	1	1	2	4	2	3	0	0	64,52	21,65	19,78	49,67	77	49,67	14,78	3,51	
**FLU-103**	2018	5	1	1	2	10	1	1	1	2	0	0	5	2	1	1	1	1	3	1	2	1	0	65,4	20,87	23,31	50,93	78	50,93	16,56	3,82	
**FLU-104**	2018	5	1	1	2	10	1	1	1	2	0	0	1	2	2	1	1	1	3	1	2	0	0	47,64	14,52	13,24	40,6	85	40,60	11,90	2,96	
**FLU-105**	2018	7	1	1	2	10	1	3	2	2	0	3	2	4	1	1	2	1	4	1	1	0	0	45,36	18,93	7,98	29,82	66	29,82	9,36	2,13	
**FLU-105-CF2**	2018	7	1	1	2	10	1	3	2	2	0	5	4	2	3	1	1	1	2	3	1	0	1	45,36	18,93	7,98	0	0				
**FLU-106**	2018	4	1	1	2	10	1	1	1	2	0	5	4	3	2	1	1	1	1	3	1	0	1	48,95	16,36	9,46	0	0				
**FLU-107**	2018	3	1	1	2	10	1	3	2	1	0	0	1	2	3	1	1	0	0	2	2	1	0	58,49	13,38	12,48	27,95	48	27,95	9,72	2,21	
**FLU-107-CF2**	2018	3	1	1	2	10	1	3	2	1	1	3	2	0	0	0	0	2	4	4	2	0	0	58,49	13,38	12,48	0	0				
**FLU-107-CF3**	2018	3	1	1	2	10	1	3	2	2	0	5	4	4	1	1	1	0	0	3	1	0	1	58,49	13,38	12,48	0	0				
**FLU-108**	2018	5	1	1	2	21	1	1	1	1	0	2	2	2	1	1	2	2	3	3	3	1	2	54,83	21,7	19,56	30	55				
**FLU-109**	2018	6	1	1	2	22	1	2	1	1	1	3	2	4	2	2	2	1	3	1	1	0	2	58,04	20,54	13,7	26,33	45	26,33	10,36	2,19	
**FLU-109-CF2**	2018	6	1	1	2	22	1	2	1	1	0	5	4	0	0	0	0	1	4	3	3	0	1	58,04	20,54	13,7	0	0				
**FLU-110**	2018	16	0	1	2	22	1	1	1	1	0	0	1	2	2	2	1	1	3	1	2	0	0	43,88	14,59	16,52	35,04	80	35,04	12,50	2,95	
**FLU-111**	2018	3	1	1	2	22	1	1	1	2	0	0	5	1	2	1	2	1	3	4	2	0	0	51,13	20,6	9,73	35,34	69	35,34	12,39	3,30	
**FLU-112**	2018	3	1	1	2	22	1	2	2	1	0	0	1	3	3	1	1	1	3	2	1	0	0	54,64	23,03	14,59	31,29	57	31,29	12,74	3,09	
**FLU-112-CF2**	2018	3	1	1	2	22	1	2	2	1	0	2	2	2	2	2	1	1	3	3	1	1	2	54,64	23,03	14,59	24,02	44				
**FLU-113**	2018	8	1	1	2	22	1	1	1	1	0	2	2	2	1	1	2	1	4	1	1	1	2	45,12	20,41	9,45	27,13	60				
**FLU-114**	2018	3	1	1	2	22	1	1	1	1	0	0	1	4	1	1	1	1	3	1	1	0	0	67,82	17,66	9,83	32,8	48	32,80	11,65	3,00	
**FLU-115**	2018	17	1	1	2	23	1	3	2	1	0	2	2	2	1	1	1	1	3	3	2	0	2	69,67	15,81	13,73	30,13	43				
**FLU-115-CF2**	2018	17	1	1	2	23	1	3	2	2	0	2	2	2	2	2	1	0	0	3	2	0	2	69,67	15,81	13,73	21,23	30				
**FLU-116**	2018	5	1	1	2	23	1	1	1	4	0	0	1	4	2	1	1	1	3	2	1	0	0	56,68	18,93	11,32	35,09	62	35,09	11,50	1,85	
**FLU-117**	2018	5	1	1	2	23	1	1	1	3	0	0	1	2	2	2	1	1	3	1	1	0	0	51,93	23,85	10,73	45,2	87	45,20	12,77	2,20	
**FLU-118**	2018	5	1	1	2	23	1	1	1	2	0	0	2	1	1	2	1	1	4	1	2	0	0	65,5	17,81	11,61	34	52	33,98	10,71	1,60	
**FLU-119**	2018	12	1	1	2	23	1	1	1	5	0	5	4	2	2	1	1	1	3	1	1	0	1	82,46	20,28	12,88	0	0				
**FLU-120**	2018	5	1	1	2	23	1	1	1	2	0	0	1	1	1	1	1	1	3	1	2	0	0	55,88	17,17	9,45	38,75	69	38,73	10,61	2,43	
**FLU-121**	2018	3	1	1	2	23	1	3	2	1	0	0	1											61,76	19,69	13,03		0				direct percussion copper
**FLU-121-CF2**	2018	3	1	1	2	23	1	3	2	2	0	0	1	2	2	2	1	1	4	4	2	0	0	61,76	19,69	13,03	47,17	76	47,15	12,61	2,31	
**FLU-122**	2018	3	1	1	2	23	1	1	1	2	0	0	1	1	3	2	1	1	3	1	1	0	0	37,96	16,2	10,51	23,6	62	23,64	7,23	1,70	
**FLU-123**	2018	5	1	1	2	23	1	1	1	2	0	0	1	4	2	1	1	1	4	4	2	0	0	55,63	21,44	11,91	32,16	58	31,79	11,82	1,87	
**FLU-124**	2018	15	1	1	2	24	1	1	1	3	0	5	4	0	0	0	2	2	4	3	2	0	1	51,18	20,34	11,16	0	0				

**FLU-125**	2018	15	1	1	2	24	1	1	1	2	0	5	4	4	2	1	1	2	4	3	2	0	1	57,52	19,23	11,25	0	0				
**3 direct percussion using antler**																																
**FLU-46**	2013	11	1	1	3	12	1	2	1	2	4	2	2	0	0	0	0	1	3	1	2	0	3	70,6	18,3	12,8	32,3	46				triple fluting
**FLU-46-CF2**	2013	11	1	1	3	12	1	2	1	1	0	1	2	1	3	2	1	0	0	3	2	1		70,6	18,3	12,8	20,8	29				
**FLU-46-CF3**	2013	11	1	1	3	12	1	2	1	1	0	1	2	4	2	1	1	0	0	1	3	1		70,6	18,3	12,8	15,4	22				
**FLU-47**	2013	12	1	1	3	12	1	1	1	0	0	2	5	1	3	2	1	1	3	4	2	0	2	84,1	19,9	9,2	56,7	67	56,72	18,56	5,08	
**FLU-48**	2013	3	1		3	13	1	2	1	1	0	1	2	3	2	2	1	1	3	2	1	0	3	40,7	12,6	5,2	14	34				double fluting
**FLU-48-CF2**	2013	3	1		3	13	1	2	1	2	0	2	2	4	2	2	1	1	3	1	1	0		40,7	12,6	5,2	20,5	50				
**FLU-49**	25/10/2013	13	1	1	3	13	1	1	1	0	0	2	5	3	2	2	1	2	3	4	3	0	2	72,6	17,5	10,7	51,2	71	51,90	15,50	2,96	
**FLU-50**	2015	3	1	1	3	14	1	4	1	1	4	2	2	1	2	2	1	0	0	2	2	1	0	60,4	23,6	13	43,8	73	43,75	16,00	3,62	triple fluting
**FLU-50-CF2**	2015	3	1	1	3	14	1	4	1	1	0	0	1	1	2	2	1	1	4	1	2	0		60,4	23,6	13	35,9	59	33,82	12,93	2,22	
**FLU-50-CF3**	2015	3	1	1	3	14	1	4	1	1	0	3	2	4	3	2	1	0	0	1	2	1		60,4	23,6	13	47,3	78	47,26	14,07	2,88	
**FLU-51**	2015	2	1	1	3	13	1	1	1	0	1	3	5	0	0	0	0	3	3	4	1	0	2	47,8	17,9	8,9	37,3	78	40,35	12,72	2,59	
**FLU-52**	2015	6	1	1	3	14	1	2	1	1	4	2	2	0	0	0	0	1	3	2	2	0	2	70,2	23,7	19,8	37,1	53				double fluting
**FLU-52-CF2**	2015	6	1	1	3	14	1	2	1	1	0	5	4	2	1	2	1	0	0	3	3	2	1	70,2	23,7	19,8	0	0				
**FLU-53**	2015	3	1	1	3	14	1	2	1	0	4	1	2	0	0	0	1	1	3	3	1	0	2	70,15	27,9	14,3	13,6	19				double fluting
**FLU-53-CF2**	2015	3	1	1	3	14	1	2	1	1	0	0	2	2	1	2	1	2	4	4	3	0		70,15	27,9	14,3	53	76	53,55	17,45	2,54	
**FLU-54**	2015	5	1	1	3	14	1	1	1	0	0	2	5	2	2	1	1	2	3	4	2	0	2	55,7	21,5	9,8	38,2	69				
**FLU-55**	2015	2	1	1	3	14	1	1	1	2	0	2&7	2	2	1	2	1	1	3	3	1	0	5	75,8	25,6	13,9	0	0				
**FLU-56**	2015	3	1	1	3	13	1	2	1	1	4	2	2	0	0	0	0	0	0	2	1	0	2	57,2	16,3	7,7	31,2	55				double fluting
**FLU-56-CF2**	2015	3	1	1	3	13	1	2	1	1	0	5	4	1	2	2	2	0	0	3	1	0	1	57,2	16,3	7,7	0	0				
**FLU-57**	2015	3	1	1	3	13	1	2	1	1	1	2	2	4	2	2	1	1	3	1	3	0	0	43,5	13,4	7	29,2	67				double fluting
**FLU-57-CF2**	2015	3	1	1	3	13	1	2	1	1	0	0	1	0	0	0	1	0	0	1	1	0		43,5	13,4	7	20,5	47				
**FLU-126**	2018	11	1	1	3	25	1	3	2	1	0	0	1	2	2	2	1	1	3	4	2	0	0	52,51	21,53	11,33	30,93	59	30,98	12,90	2,65	
**FLU-126-CF2**	2018	11	1	1	3	25	1	3	2	2	0	0	1	3	2	2	1	1	3	1	1	0	0	52,51	21,53	11,33	38,53	73	38,53	12,07	2,65	
**FLU-127**	2018	3	1	1	3	25	1	3	2	1	0	3	2	1	2	2	1	2	4	1	2	0	2	56,05	16,54	11,36	24	43				
**FLU-127-CF2**	2018	3	1	1	3	25	1	3	2	1	0	2	2	1	2	2	1	1	3	1	1	0	2	56,05	16,54	11,36	39,8	71				
**FLU-128**	2018	13	1	1	3	25	1	1	1	1	0	2	2	2	3	2	1	0	0	3	3	1	2	60,64	20,42	11,61	27,37	45				
**FLU-129**	2018	2	1	1	3	26	1	1	1	1	0	0	1	4	2	2	1	2	4	4	3	0	0	42,8	16,72	8,88	28,9	68	28,90	10,78	2,12	
**FLU-130**	2018	5	1	1	3	26	1	1	1	1	0	2	1	2	3	2	1	2	4	3	3	1	2	50	20,04	14,65	32,33	65				
**FLU-131**	2018	5	1	1	3	27	1	1	1	2	0	3	1	2	1	1	1	2	3	1	2	0	2	59,01	17,36	11,76	40,62	69				
**FLU-132**	2018	4	1	1	3	27	1	2	2	1	0	0	1	2	2	1	1	1	3	2	2	0	0	62,12	20,92	14,58	33,49	54	33,47	11,94	2,28	
**FLU-132-CF2**	2018	4	1	1	3	27	1	2	2	1	0	0	5	2	3	2	1	0	0	4	3	1	2	62,12	20,92	14,58	47,2	76				
**FLU-133**	2018	3	1	1	3	27	1	2	2	1	0	0	1	2	1	1	1	1	3	1	1	0	0	51,87	24,49	12,59	34,89	67	34,89	10,46	2,36	
**FLU-133-CF2**	2018	3	1	1	3	27	1	2	2	2	0	1	2	2	2	2	1	0	0	1	1	0	2	51,87	24,49	12,59	17,94	35				
**FLU-134**	2018	5	1	1	3	27	1	1	1	1	0	0	1	1	1	2	1	1	4	2	2	0	0	69,11	20,11	12,96	51,26	74	51,26	12,24	2,89	
**FLU-135**	2018	3	1	1	3	27	1	3	2	3	0	0	1	3	3	1	1	1	3	1	2	0	0	46,05	18,73	10,17	32,38	70	32,38	8,67	2,41	
**FLU-135-CF2**	2018	3	1	1	3	27	1	3	2	3	0	0	1	1	2	2	1	1	3	1	2	0	0	46,05	18,73	10,17	39,9	87	39,90	10,65	2,51	
FLU-136	2018	3	1	1	3	27	1	2	2	1	0	0	1											62,28	20,52	11,56		0				direct percussion copper
**FLU-136-CF2**	2018	3	1	1	3	27	1	2	2	2	0	0	1	2	2	2	1	2	4	2	2	1	0	62,28	20,52	11,56	42,85	69	42,85	12,67	2,36	
**FLU-137**	2018	3	1	1	3	27	1	3	2	3	0	0	1	1	2	1	1	1	3	1	2	0	0	60,49	26,32	11,37	25,91	43	25,91	15,14	1,65	
**FLU-137-CF2**	2018	3	1	1	3	27	1	3	2	1	0	0	1	2	2	2	1	1	3	1	2	0	0	60,49	26,32	11,37	40,47	67	40,45	12,94	4,36	
**FLU-138**	2018	6	1	1	3	27	1	1	1	3	0	0	1	2	2	2	1	1	3	1	1	0	0	60,48	22,36	9,58	31,82	53	31,82	8,53	3,17	
**FLU-139**	2018	5	1	1	3	27	1	2	2	1	0	0	1	2	2	2	1	1	3	1	1	0	0	42,87	11,99	7,55	23,51	55	23,51	6,35	2,32	
**FLU-139-CF2**	2018	5	1	1	3	27	1	2	2	1	0	5	4	1	2	2	1	2	4	3	3	0	1	42,87	11,99	7,55	0	0				
**FLU-140**	2018	5	1	1	3	27	1	3	2	2	0	0	1	4	2	1	1	1	3	2	1	0	0	61,78	21,38	10,61	26,36	43	26,36	9,07	2,54	
**FLU-140-CF2**	2018	5	1	1	3	27	1	3	2	2	0	0	1	2	1	2	1	1	3	2	1	0	0	61,78	21,38	10,61	45,34	73	45,34	11,41	2,96	
**FLU-141**	2018	8	1	1	3	27	1	3	2	1	0	0	1	3	2	2	1	1	3	2	1	0	0	47,6	14,81	9,03	26,12	55	26,12	7,32	1,34	
**FLU-141-CF2**	2018	8	1	1	3	27	1	3	2	3	0	0	1	2	2	2	1	1	3	4	2	0	0	47,6	14,81	9,03	31,37	66	31,37	13,08	2,86	
**4 direct percussion using hard wood**																																
**FLU-58**	2014	5	1	1	4	16	1	1	1	0	0	3	5	1	2	2	1	2	4	3	2	0	2	68,8	17,4	10,9	52,2	76	52,78	13,29	2,86	

**FLU-59**	2014	3	1	1	4	16	1	3	1	1	4	5	4	0	0	0	0	1	2	3	1	0	2	83,9	17,8	8,6	25,1	30				double fluting
**FLU-59-CF2**	2014	3	1	1	4	16	1	3	1	0	4	1	2	0	0	0	0	1	3	1	2	0	1	83,9	17,8	8,6	0	0				
**FLU-60**	2015	3	1	1	4	16	1	1	1	4	0	5	4	1	2	1	1	0	0	3	1	0	6	68,5	22,8	10,4	0	0				
**FLU-61**	2015	5	1	1	4	16	1	1	1	2	0	2	2	1	2	2	1	1	3	1	3	1	2	60,2	16,6	9,4	29	48				
**FLU-62**	2015	13	1	1	4	16	1	2	1	1	0	1	2	1	2	2	1	2	4	4	3	1	2	60,2	22,8	13,4	21,7	36				double fluting
**FLU-62-CF2**	2015	13	1	1	4	16	1	2	1	2	0	5	4	2	1	2	1	3	4	1	2	0	1	60,2	22,8	13,4	0	0				
**FLU-63**	2015	8	1	1	4	16	1	1	1	2	0	2	2	2	2	2	1	1	2	1	2	0	2	49	20	8,3	34	69				
**FLU-64**	2015	14	0	1	4	16	1	2	1	1	0	2	2	2	2	2	1	1	2	3	3	0	0	48,7	22,4	15,7	29,2	60				double fluting
**FLU-64-CF2**	2015	14	0	1	4	16	1	2	1	2	1	0	1	2	2	2	1	1	2	3	3	0		48,7	22,4	15,7	23,15	48				
**FLU-65**	2015	14	0	1	4	16	1	2	1	0	4	2	2	0	0	0	0	1	3	1	3	0	0	49	23,5	12,7	19,7	40				double fluting
**FLU-65-CF2**	2015	14	0	1	4	16	1	2	1	1	0	0	1	2	3	2	1	1	3	1	3	0		49	23,5	12,7	32,7	67				
**FLU-66**	2015	14	0	1	4	16	1	1	1	2	0	5	4	1	1	2	1	0	0	3	2	0	1	43,7	20	17	0	0				
**FLU-67**	10/03/2015	14	0	1	4	16	1	2	1	0	4	1	2	0	0	0	0	2	3	3	3	0	3	59	24,7	13,6	15,6	26				double fluting
**FLU-67-CF2**	11/03/2015	14	0	1	4	16	1	2	1	1	0	2	2	1	2	2	1	1	4	3	3	1	3	59	24,7	13,6	25,7	44				
**FLU-68**	10/03/2015	14	0	1	4	16	1	1	1	0	0	5	4	1	2	2	1	1	3	3	3	1	1	52,2	19,2	11,6	0	0				
**FLU-69**	20/04/2015	14	0	1	4	16	1	1	1	2	1	3	2	0	0	0	0	2	3	1	3	0	2	69	24,1	10,7	39,4	57				
**FLU-70**	06/05/2015	14	0	1	4	16	1	1	1	2	0	3	2	2	3	2	1	2	3	3	3	0	2	63,8	25,2	12,5	44	69				
**FLU-71**	12/05/2015	5	1	1	4	16	1	1	1	2	0	5	4	2	2	2	1	3	3	3	2	0	1	75,8	21,6	19,7	0	0				
**FLU-72**	12/05/2015	5	1	1	4	16	1	3	1	2	0	7	2	1	2	2	1	1	3	3	3	0	0	52,6	18	19,2	44,4	84	44,09	14,07	2,31	
**FLU-73**	13/05/2015	15	1	1	4	16	1	3	1	2	0	2	2	4	1	2	1	0	0	1	3	0	3	55,9	19,1	13,2	28,4	51	44,07	10,11	2,11	double fluting
**FLU-73-CF2**	13/05/2016	15	1	1	4	16	1	3	1	1	1	3	2	0	0	0	0	1	3	1	2	0		55,9	19,1	13,2	42,3	76				
**FLU-74**	2015	3	1	1	4	16	1	1	1	0	0	0	1	4	1	2	1	2	3	1	2	0	0	64	25,7	16,9	56,7	89	56,80	14,45	2,31	
**FLU-75**	2015	3	1	1	4	16	1	1	1	2	0	4	4	2	2	2	1	2	2	1	3	0	0	72,38	35	19	66,12	91	72,40	20,02	3,62	
**FLU-76**	2015	5	1	1	4	16	1	1	1	2	0	3	5	2	1	2	1	2	4	4	2	0	2	102,4	26,8	21,4	83,9	82	84,86	20,27	4,21	
**FLU-77**	2015	3	1	1	4	18	1	2	1	1	0	0	1	1	2	2	1	1	2	2	2	0	2	95,2	28,8	14,5	80,3	84	79,40	17,80	2,57	double fluting
**FLU-77-CF2**	2015	3	1	1	4	18	1	2	1	4	4	3	2	0	0	0	0	2	3	4	3	0		95,2	28,8	14,5	63,8	67	64,12	18,56	3,46	
**FLU-78**	2015	6	1	1	4	17	1	2	1	1	0	1	2	2	1	1	1	0	0	3	2	2	2	63,1	25,4	16,1	22,6	36				double fluting
**FLU-78-CF2**	2015	6	1	1	4	17	1	2	1	2	3	0	1	1	1	2	1	2	2	1	2	0		63,1	25,4	16,1	46	73				
**FLU-79**	2015	5	1	1	4	16	1	2	1	1	1	2	2	0	0	0	0	1	3	3	3	0	2	64	19,3	12	33,9	53				double fluting
**FLU-79-CF2**	2015	5	1	1	4	16	1	2	1	2	1	0	1	0	0	0	0	1	4	1	2	0		64	19,3	12	32,4	51				
**FLU-80**	2015	3	1	1	4	16	1	2	1	0	4	1	2	0	0	0	0	0	0	3	3	0	3	63,2	24,2	10,5	16,6	26				double fluting
**FLU-80-CF2**	2015	3	1	1	4	16	1	2	1	2	4	2	1	0	0	0	0	2	3	3	2	0		63,2	24,2	10,5	39,3	62				
**FLU-81**	2015	3	1	1	4	16	1	1	1	2	0	3	2	1	2	2	2	0	0	1	2	0	2	57,3	20,4	15,07	38	66				
**FLU-82**	2015	1	0	1	4	19	1	2	1	0	4	1	2	0	0	0	0	1	2	3	1	1	2	72,4	24,5	14,7	17,8	25				double fluting
**FLU-82-CF2**	2015	1	0	1	4	19	1	2	1	2	4	0	1	1	2	2	1	2	3	1	3	0		72,4	24,5	14,7	55,9	77				
**FLU-83**	2015	6	1	1	4	16	1	1	1	4	0	4	4	4	2	2	1	2	4	1	2	0	1	68,2	22,2	12,2	0	0	56,37	12,70	1,95	
**FLU-84**	2015	6	1	1	4	19	1	1	1	3	0	5	4	1	2	2	1	1	3	1	2	0	1	63,7	18	12,1	0	0				proximal trihedral shaping
**FLU-85**	2015	8	1	1	4	17	1	2	1	1	0	2	5	1	1	2	1	1	4	3	3	0	3	70	21,2	11,16	39,9	57				double fluting_failure: wood too dry
**FLU-85-CF2**	2015	8	1	1	4	17	1	2	1	3	0	3	2	1	2	2	1	2	4	3	3	0		70	21,2	11,16	45,9	66				
**FLU-86**	2015	8	1	1	4	16	1	1	1	3	0	5	4	2	1	2	1	3	4	3	2	0	1	88,8	23,4	17,4	0	0				
**FLU-87**	2015	6	1	1	4	16	1	1	1	0	0	3	2	1	3	2	1	2	4	4	2	0	2	53,5	19,3	11,7	36,4	68				failure: wood too dry
**FLU-88**	2015	5	1	1	4	16	1	1	1	3	0	3	2	2	1	3	1	3	3	2	3	0	2	74,6	23,3	22,8	56,9	76				failure: wood too dry
**FLU-89**	2015	8	1	1	4	16	1	1	1	3	0	5	4	1	2	2	2	1	2	3	1	0	1	79,2	25,5	14,6	0	0				failure: wood too dry
**FLU-90**	2015	3	1	1	4	20	1	4	1	3	0	0	1	1	1	2	1	2	3	2	3	0	2	63,6	28,8	10,7	57,6	91	59,50	10,35	1,77	quadruple fluting
**FLU-90-CF2**	2015	3	1	1	4	20	1	4	1	1	4	2	2	1	1	2	1	1	3	1	2	0		63,6	28,8	10,7	37,1	58				
**FLU-90-CF3**	2015	3	1	1	4	19	1	4	1	3	0	0	1	0	0	0	0	2	4	1	3	1		63,6	28,8	10,7	63,6	100	63,26	15,30	2,77	
**FLU-90-CF4**	2015	3	1	1	4	16	1	4	1	3	0	3	2	1	2	2	1	2	3	4	2	0		63,6	28,8	10,7	62	97	57,19	14,67	2,75	
**FLU-91**	2015	3	1	1	4	16	1	1	1	2	3	3	2	2	1	2	1	2	3	2	1	0	2	58,6	18,7	8,2	43,8	75	45,06	8,83	2,06	
**FLU-92**	2015	3	1	1	4	16	1	1	1	2	0	2	2	1	2	2	1	1	4	4	2	0	2	56,4	19,4	10,5	37,7	67	37,10	11,56,	1,40	
**FLU-93**	2015	6	1	1	4	15	1	2	1	2	1	2	2	0	0	0	0	2	3	3	2	0	3	70	22,5	12,9	31,1	44				double fluting
**FLU-93-CF2**	2015	6	1	1	4	15	1	2	1	1	0	2	2	1	1	2	1	2	3	1	2	0		70	22,5	12,9	33,3	48				

**FLU-94**	2015	6	1	1	4	15	1	1	1	3	0	5	4	1	1	2	1	1	2	3	1	0	1	56,8	23	12,7	0	0				
**FLU-95**	2015	2	1	1	4	15	1	1	1	2	0	3	2	2	2	2	1	3	3	4	3	0	2	61,4	21,8	10,4	44	72				
**FLU-96**	2018	5	1	1	4	16	1	1	1	2	3	0	1	1	2	2	0	1	2	1	1	0	0	63,6	17,4	11,4	31,8	50				
**FLU-97**	2018	5	1	1	4	16	1	1	1	1	0	0	1	1	1	2	0	1	1	1	2	0	0	68,8	20,4	10	41,6	60	40,37	10,84	1,73	
**FLU-98**	2018	2	1	1	4	16	1	2	1	1	0	1	2	1	1	2	0	0	0	1	1	0	3	49,6	15,2	10,4	8,3	17				
**FLU-98-CF2**	2018	2	1	1	4	16	1	2	1	2	1	3	2	0	0	0	0	1	3	1	1	0	2	49,6	15,2	10,4	33	67				
**5 indirect percussion using antler**																																
**FLU-99**	2015	1	0	1	5	4	5	1	1	5	0	4	4	1	2	2	1	1	1	1	1	0	1	40	19,4	8,34	0	0				
**FLU-100**	2015	7	1	1	5	4	5	1	1	3	0	4	4	1	3	2	1	0	0	1	1	0	0	50,3	21,2	6	50,1	100	49,04	11,06	1,90	
**FLU-142**	2018	15	1	1	5	28	5	2	2	1	0	0	1	1	3	2	1	0	0	1	2	0	0	50,9	22,14	9,8	29,16	57	29,17	9,64	2,14	
**FLU-142-CF2**	2018	15	1	1	5	28	5	2	2	1	0	0	1	2	2	1	1	0	0	1	1	0	0	50,9	22,14	9,8	28,89	57	29,00	9,07	1,89	
**FLU-143**	2018	12	1	1	5	28	5	1	1	3	0	4	3	2	2	1	1	2	4	1	1	0	1	47,69	15,65	8,14	0	0				
**FLU-144**	2018	6	1	1	5	28	5	2	2	1	0	0	1	2	3	2	1	0	0	2	1	0	0	59,61	17,11	8,02	24,33	41	24,33	9,70	1,77	
**FLU-144-CF2**	2018	6	1	1	5	28	5	2	2	5	0	0	1	1	2	1	1	1	4	1	1	0	0	59,61	17,11	8,02	59,61	100	60,00	10,63	2,56	
**FLU-145**	2018	18	0	1	5	28	5	1	1	2	0	3	2	1	1	1	2	1	4	1	2	1	0	45,19	14,42	9,73	28,38	63	28,38	7,94	2,23	
**FLU-146**	2018	19	1	1	5	28	5	1	1	1	0	0	1	2	1	2	1	1	2	2	1	0	0	52,02	17,06	8,99	44,52	86	44,52	10,96	2,65	
**FLU-147**	2018	8	1	1	5	28	5	1	1	5	0	0	1	2	2	2	1	1	4	1	2	0	0	64,14	19,94	13,11	64,14	100	64,10	13,95	3,06	
**FLU-148**	2018	5	1	1	5	28	5	1	1	2	0	5	4	2	2	2	1	0	0	1	1	0	1	56,76	18,32	8,55	0	0				
**FLU-149**	2018	5	1	1	5	28	5	1	1	5	0	5	4	2	3	1	1	1	3	1	1	0	1	57,95	17,43	7,74	0	0				
**FLU-150**	2018	6	1	1	5	28	5	1	1	1	0	5	4	2	2	2	1	0	0	1	1	0	1	51,11	19,38	11,6	0	0				
**FLU-151**	2018	3	1	1	5	28	5	1	1	2	0	0	1	1	2	2	1	1	3	1	1	0	0	52,41	16,71	9,38	52,41	100	52,41	11,15	2,24	
**FLU-152**	2018	5	1	1	5	28	5	1	1	2	0	0	1	1	2	2	1	1	3	1	1	0	0	51,84	16,45	7,05	51,84	100	51,80	10,36	2,98	
**FLU-153**	2018	8	1	1	5	28	5	1	1	1	0	0	1	2	2	1	1	0	0	2	1	0	0	46,31	16,54	11,41	29,63	64	30,00	7,76	2,03	
**FLU-154**	2018	5	1	1	5	28	5	1	1	8	0	4	3	2	2	1	1	1	4	1	2	0	0	54,08	16,12	10,76	54,08	100	54,00	10,15	2,79	
**FLU-155**	2018	20	1	1	5	28	5	1	1	3	0	4	3	2	2	2	1	1	4	1	3	0	0	52,26	17,57	7,53	52,26	100	52,26	10,40	2,28	
**FLU-156**	2018	5	1	1	5	28	5	1	1	4	0	5	4	2	2	2	1	0	0	1	1	0	1	53,17	11,86	9,53	0	0				
**FLU-157**	2018	6	1	1	5	28	5	1	1	2	0	0	1	2	1	1	1	1	3	2	1	0	0	52,75	16,5	9,96	34,59	66	34,59	7,87	2,35	
**FLU-158**	2018	5	1	1	5	28	5	1	1	1	0	0	1	2	2	2	1	1	4	1	1	0	0	57,65	18,71	10,58	56,1	97	56,10	10,34	2,54	
**FLU-159**	2018	4	1	1	5	28	5	1	1	3	0	4	3	1	2	1	1	1	4	1	1	0	0	56,61	17,51	13,34	56,61	100	56,61	9,28	2,26	
**FLU-160**	2018	1	1	1	5	28	5	1	1	3	0	3	2	2	2	1	1	1	3	1	1	0	0	75,37	16,9	12,44	40,07	53	40,07	10,18	2,75	

S3 Table (continued). Experimental corpus of fluted points used in this study. Below, are explained the codes in S3_Table

Raw material (R.M.)	**1.** Eocene resinite opal (49), **2.** Eocene flint (79), **3.** Bajocian flint (16), **4.** Eocene flint (86), **5.** Turonian flint (37), **6.** Turonian flint (17), **7.** Coniacian flint (76), **8.** Maastrichtian flint (24), **9.** Cenomanian flint (76), **10.** Chalcedony (29), **11.** Turonian flint (16), **12.** Bathonian flint (86), **13.** Oligocene flint (30), **14.** Miocene flint (41), **15.** Bajocian-Bathonian flint (86), **16.** Radiolarite (Masirah-Oman), **17.** Chalcedony (Suhila-U.A.E), **18.** Eocene flint (Shuwaymyah -Oman), **19.** Eocene flint (Duqm - Oman), **20.** Turonian flint (45)
Heat treatment (H.T.)	**0** heat treatment **1** no heat treatment
Shaping (Shap.)	**1** direct soft hammer percussion and pressure **2** other
Fluting technique (Fl. T)	**1** pressure **2** direct percussion using soft stone **3** direct percussion using antler **4** direct percussion using hard wood **5** indirect percussion using antler
Tool	**1.** Antler stick (*Cervus elaphus* , 23 grams), **2.** Short crutch with antler point (*Cervus elaphus,* 170 grams) , **3.** antler tine (*Cervus elaphus,* 73 grams), **4.** Short antler curved punch (*Cervus elaphus,* 30 grams) and hardwood hammer (*Buxus semperviren,* 300 grams), **5.** Soft stone hammer (limestone, 150 grams), **6.** Soft stone hammer (micritic limestone, 145 grams), **7.** Soft stone hammer (micritic limestone, 245 grams), **8.** Soft stone hammer (micritic limestone, 213 grams), **9.** Soft stone hammer (fine limestone, 116 grams), **10.** Soft stone hammer (trachyte, 128 grams), **11.** Soft stone hammer (fine limestone, 184 grams), **12.** Antler hammer (*Odocoileu virginianus,* 240 grams), **13.** Antler hammer (*Alces alce,* 111 grams), **14.** Antler hammer (*Cervus elaphus,* 262 grams), **15.** Hardwood Hammer (*Buxus sempervirens,* 200 grams), **16.** Hardwood hammer (*Buxus sempervirens,* 290 grams), **17.** Hardwood hammer (*Buxus sempervirens,* 350 grams), **18.** Hardwood hammer (*Buxus sempervirens,* 450 grams), **19.** Hardwood hammer (*Cornus mas,* 285 grammes), **20.** Hardwood hammer (*Manilkara sp.,* 300 grams), **21.** Soft stone hammer (marble, 224 grams), **22.** Soft stone hammer (marble, 136 grams), **23.** Soft stone hammer (soft sandstone, 114 grams), **24.** Soft stone hammer (sandstone, 130 grams), **25.** Antler hammer (Alces alce,190 grams), **26.** Antler hammer (*Alces alce* ,105 grams), **27.** Antler hammer (*Alces alce* , 66 grams), **28.** Short antler straight punch (*Capreolus Capreolus* , 25 grams) and hardwood hammer (*Buxus semperviren* , 245 grams)
Holding (Hold.)	**1** free hand **2** free hand on wooden support **3** pressure Pelegrin mode2 **4** pressure small lever **5** wooden grooved support
Fluting organisation flûtage (Fl. Org.)	**1** unifacial unipolar **2** unifacial bipolar **3** unifacial unipolar multiple **4** unifacial bipolar multiple
Number of channel-flake (C-F nbr.)	n
Number of fragments (Fgts. nbr.)	**0** whole **n** number of fragments
Lack of parts (Lack)	**0** none **1** proximal **2** mesial **3** distal **4** multiple
Channel-flake accidents (C-F Acc.)	**0** none **1** hinged proximal **2** hinged mesial **3** hinged distal **4** slight distal plunging **5** major plunging **6** edge pulling **7** double flaking
Channel-flake distal profile (C-F dist. prof.)	**1** normal **2** hinged **3** slightly plunged **4** plunged **5** overthickness and distal enlargement
Butt (facetted-convex-abraded)	**0** unobservable **1** narrow **2** large **3** punctiform **4** linear
Bulb	**0** unobservable **1** almost nonexistent **2** slighlty pronounced **3** pronounced
Lip	**0** unobservable **1** absent **2** present **3** developed
Bulb scars (Bb. sc.)	**0** unobservable **1** absent **2** present
Ripples amplitude on channel-flake (Rip. Am. C-F)	**0** absent **1** very light **2** pronounced **3** well pronounced
Ripples localisation on channel-flake (Rip. Loc. C-F)	**0** absent **1** proximal part **2** mesial part **3** distal part **4** mesial and distal part
Edges parallelism on channel-flake (Edg. Par. C-F)	**1** parallel **2** convergent **3** divergent **4** divergent 2/3 and convergent distal end
Edges regularity on channel-flake (Edg. Reg. CF)	**1** straight **2** sinuous **3** irregular
Channel-flake overflowing (C-F ov.fl.)	**0** non overflowing **1** overflowing one side **2** overflowing two sides
Fluted piece accidents (F.P Acc.)	**0** no prohibitive accidents **1** plunging fracture **2** major hinged fracture simple **3** major hinged fracture double **4** distal counterblow **5** flexion breakage **6** plunging and flexion breakage **7** hinged and distal counterblow
Maximum lenght fluted piece (l. F.P) - mm	n mm
Maximum width fluted piece (w. F.P) - mm	n mm
Maximum thickness fluted piece (th. F.P) - mm	n mm
Fluting lenght (Fl. l.) - mm	n mm
% fluted lenght ([Fl. l./l. F.P]x100)	n%
Maximum lenght channel-flake (l. C-F) - mm	n mm
Maximum width channel-flake (L. C-F)- mm	n mm
Maximum thickness channel-flake (Ep. C-F) - mm	n mm
Observations - remarks	
